# Abstracts of the 2023 49th Annual NATAS Conference

**DOI:** 10.3390/polym15153250

**Published:** 2023-07-30

**Authors:** David LaVan, Feng Yi, Tina Adams, Ran Tao, Elizabeth Pelczar, Han Xia, Xiao Hu, Stephen Sauerbrunn, Janis Matisons

**Affiliations:** 1 National Institute of Standards and Technology, Gaithersburg, MD 20899, USA; 2 The Lubrizol Corporation, Wickliffe, OH 44092, USA; tina.adams@lubrizol.com; 3 New Jersey Department of Public Health, Ewing, NJ 08628, USA; elizabeth.pelczar@doh.nj.gov; 4 Eli Lilly and Co., Indianapolis, IN 46285, USA; hxia@lilly.com; 5 Department of Physics and Astronomy, College of Science and Mathematics, Rowan University, Glassboro, NJ 08028, USA; hu@rowan.edu; 6 Center for Composite Materials, University of Delaware, Newark, DE 19716, USA; sauerbru@udel.edu; 7 Intertape Polymer Group Company, Marysville, MI 48040, USA; jmatison@itape.com

**Keywords:** thermal analysis, energetic materials, additive manufacturing, kinetics, polymers, nanocalorimetry, rheology

## Abstract

We are pleased to announce that the 49th annual meeting of NATAS cwill be held in Rockville, MD, a beautiful city that is part of the Washington D.C. National Capital Area, on the DC metro system (allowing easy access to Washington museums and sites), and close to the National Institute of Standards and Technology (NIST), National Institutes of Health (NIH), Naval Research Laboratory (NRL), Army Research Laboratory (ARL), Johns Hopkins University Applied Physics Laboratory (JHU-APL), Naval Surface Warfare Center (NSWC), Georgetown University, George Washington University, George Mason University, and the University of Maryland. The North American Thermal Analysis Society is a venerable organization that offers scientists and practitioners the opportunity to explore the frontiers of thermal analysis, rheology, and materials characterization. The NATAS meeting always features new developments in the science of thermal analysis as well as applications of these techniques in a wide variety of fields. The meeting includes exhibits from vendors allowing for personalized attention and connecting with providers of instrumentation and software related to thermal analysis. The Society appreciates the financial support of the following contributing sponsors this year: NIST, Mettler-Toledo, TA Instruments, SETARAM, Anton Paar USA, MDPI AG, TAFDV, and QΔT Lab. Exhibitors include AKTS SA, Anton Paar USA, Mettler-Toledo, McCrone Microscopes & Accessories, NETZSCH Instruments North America, TA Instruments, and Thermtest, Inc.

## Introduction

This year, there are sessions on advances in instrumentation, reaction kinetics, nanocalorimetry, rheology, thermal transport, and thermophysical properties. Other sessions are dedicated to applications in 3D printing/Additive Manufacturing, electronic packaging, energetic materials, polymers. Plenary speakers are Professor Amy Marconnet of Purdue University, Dr. Steven Choquette of the National Institute of Standards and Technolocy, Professor Rigoberto Advincula, of the University of Tennessee at Knoxville, selected as NATAS Fellow this year, and Dr. Kenneth Kearns of the Dow Chemical Company, who is the recipient of the NATAS Metter-Toledo Award in Thermal Analysis. 

## 1. Plenary Lectures

### 1.1. Thermal Metrology for Multi-Scale and Heterogeneous Systems

Amy Marconnet *

Purdue University
* Correspondence: marconnet@purdue.edu

**Abstract:** As researchers develop new materials and systems, thermal transport is often key to performance, safety, and reliability. For instance, in battery cells, interfaces and low conductivity pathways can lead to high temperatures that can lead to thermal runaway. In portable and wearable electronics, limited heat dissipation pathways lead either to temperatures that require throttling device performance or that degrade the system. Open challenges exist in optimizing and tuning the thermal transport within these heterogeneous systems, while meeting constraints on mechanical properties and device performance. Ultimately, efficient, thermally informed engineering is needed to translate research into technology and requires integrated modeling, experiments, and materials development. This talk will describe several recent examples from my group of engineering materials from the nano- and micro-structural level to achieve targeted performance objectives coupled with the development of new tools to measure their properties and performance. Additionally, we build from steady state to transient heat dissipation systems for electronics cooling and other applications, illustrating combined experimental and modeling approaches.

### 1.2. An Introduction to NIST Standard Reference Materials: Their History, Use, and Modes of Certification

Steven Choquette *

National Institute of Standards and Technology
* Correspondence: steven.choquette@nist.gov

**Abstract:** The NIST (formerly the National Bureau of Standards or NBS) is one of the oldest federal labs still in operation. The NBS was founded in 1901 to address the “challenges“ of the lack of standardized units of measure useful for commerce. Examples included eight different authoritative values for the gallon, a dearth of standards for the emerging electrical industry, poor and uneven quality of consumer products and materials, and no federal capability for calibration of test instruments. Early in the Bureau’s history, train wrecks were unfortunately all too common due to wheel and axle failures. Although the alloy composition for these parts was well specified, steel foundry chemistry laboratories lacked standards to validate their melts. Collaborative work between NBS chemists and the American Foundrymen’s Association led to the first cast iron chip standards in the form of Standard Iron A, B, C, and D. These standard samples led to improved material validation and railway safety.

Moving forward a century, NIST now supports greater than 1100 different SRMs that support accuracy in measurements in health care, food safety, biomanufacturing, the environment, forensics and, to this day, the elemental compositions of ferrous and nonferrous metals. This talk will describe the “making of the sausage“, i.e., how we prioritize developing new standards, their use by our stakeholders, and some new and exciting developments in our first living cell reference material. 

### 1.3. A Perspective on Characterization of High-Performance Polymer Materials and 3D/4D Printing

Rigoberto Advincula *

University of Tennessee at Knoxville
* Correspondence: radvincu@utk.edu

**Abstract:** The advent of 3D printing has enabled prototypes and devices of high-performance polymeric materials which have appended functionality based on composition and macromolecular design; 3D printed polymers can be further classified into thermoplastics, thermosets and elastomers based on their corresponding thermo-mechanical properties. However, using multi-materials and 4D printing allows the design of new materials and applications based on integrating conversion chemistry with the printing mode. This talk will demonstrate the importance of thermo-mechanical characterization (DSC, TGA, DMA, GC/MS Pyrolysis, etc.) and experimental design (percolation theory and learning) for high-performance and stimuli-responsive properties and 4D fabrication of multi-materials, including polyurethane, silicone, epoxy, benzoxazine thermosets, and nanocomposites with concept objects. While most of the method focuses on demonstrating DIW or viscous solution printing (VSP), other works and methods using SLA, SLS, and FDM 3D Printing have proven the importance of this focus.

### 1.4. Use of Thermal Analysis to Better Understand the Role of Processing on Material Performance: From Organic Glasses to Semi-Crystalline Polymers

Kenneth Kearns *

Dow Chemical Company
* Correspondence: klkearns@dow.com

**Abstract:** Material performance is dictated not only by chemical structure but also the processing used. This talk will focus on how thermal analysis has been a useful tool to understand the role of different processing schemes in dictating final performance for a broad range of materials. Differential scanning calorimetry enabled the discovery and development of stable, small molecule organic glasses processed via vapor deposition. Once made, understanding how these stable glasses transform becomes important. Modulated nanocalorimetry showed that these stable glasses transformed into supercooled liquids by either a surface-initiated transformation or a bulk nucleation and growth mechanism depending on glass thickness. Recently, we have used fast-scanning calorimetry (FSC) at Dow to study flow-enhanced nucleation in polyethylene. We were able to measure the overall crystallization kinetics of polyethylene ex situ, after processing; the fast heating and cooling rates of FSC relative to the relaxation timescale of polymer chains enabled these measurements. This experimental work was used by our external collaborators to make advancements towards a fully integrated processing model for semi-crystalline polyethylene, which has significant industrial relevance. 

## 2. Session: Polymers

### 2.1. Unexpected Insights from the “Basics”

Pamela Percha *

3M Company
* Correspondence: papercha@mmm.com

**Abstract:** Materials characterization taps into a variety of analytical techniques which are applied in both research and manufacturing environments. Thermal analysis, and more specifically Differential Scanning Calorimetry, is an example of a key approach applied in a wide range of situations spanning raw material screening, characterization, and validation to resolving processing inconsistencies or product failures and ultimately even Intellectual Property claims for patents. This presentation will discuss the foundation or “basics“ of thermal analysis, that is, what the various features typically “look “ like in thermal data and what the artifacts or features can reveal to scientists. 

### 2.2. Optimizing and Monitoring UV-Cure Process by DSC, DEA and DMA

Yanxi Zhang *

Netzsch Instruments North America
* Correspondence: Yanxi.zhang@netzsch.com

**Abstract:** A variety of questions may arise in the UV curing process of polymeric materials. For example, when does UV curing start and complete? What is the reactivity of the resin? What is the glass transition temperature after curing? Which photo initiator does show best performance? How do the mechanical properties of the cured material change during the UV curing process? 

Differential Scanning Calorimetry (DSC), Dielectric Analysis (DEA), and Dynamic Mechanical Analysis (DMA) offer effective means to answer these questions. DSC measures the reaction enthalpy and degree of cure initiated by radiation. DEA allows for the measurement of changes in the dielectric properties related to ion mobility and dipole alignment during curing. Compared with DSC, DEA is good for fast curing systems because the data acquisition rate is less than 5 ms and is more sensitive to small changes close to the end of curing. DMA measures modulus changes during the UV curing process. These thermal analysis methods are indispensable in both R&D and QC in the area of UV curing.

### 2.3. Influence of Moisture on Glass Transition Temperature and Mechanical Properties of Epoxy Resin Using DMA with Controlled Relative Humidity

Steve Sauerbrunn *, Sagar Doshi, Joseph Deitzel, Alex Schneider and John W. Gillespie, Jr.

University of Delaware—CCM
* Correspondence: sauerbru@udel.edu

**Abstract:** Epoxy resins are widely used in composites due to their mechanical properties, thermal stability, and relative ease of processing, and have various applications from automotive to aerospace. They are often subjected to a wide range of environmental conditions, from extreme temperatures to high relative humidity. Epoxies are known to susceptible to moisture, leading to degradation of their properties due to moisture ingress.

The effect of moisture on epoxy not only leads to deterioration of mechanical properties but to suppression of the glass transition temperature. Typically, ASTM D7028 [1] is the standard used for characterizing dry and wet Tg by DMA. However, the standard over-simplifies the challenges involved in wet Tg measurement and does not consider the heat and mass transfer mechanisms. Slower rates cannot be used for measuring wet Tg due to the drying of the specimen, and faster rates could lead to a temperature gradient in the specimen. Researchers have developed a procedure which involves the development of a diffusion model for the material along with temperature correction data using a thermocouple in the center of the specimen [2].

In this research, we use a novel piece of equipment, namely, a Dynamic Mechanical Analyzer (Netzsch DMA Eplexor) fitted with a chamber to maintain relative humidity, to study the influence of moisture on the glass transition temperature and mechanical properties of the epoxy resin SC-15. Specimens were run at ASTM recommended heating rates (5 °C/min) and a slow heating rate of 0.5–1 °C/min. Preliminary results for the tests conducted for specimens with and without controlling relative humidity at the slow heating rate indicate a shift in the onset of Tg and show evidence of loss of moisture in the Tan Delta curves; ~20 °C difference in the Tg (by Tan Delta) for specimens is observed when the relative humidity is maintained while running the DMA.

**References**

1. *ASTM D 7028*; Standard Test Method for Glass Transition Temperature (DMA Tg) of Polymer Matrix Composites by Dynamic Mechanical Analysis (DMA). ASTM International.
2. Lee, C.W.; Tienda, K.A.; Storage, T.M.; Brockman, B.; Gibson, T. Characterization of Wet-Glass-Transition Temperatures for High Temperature Polymers. In Proceedings of the SAMPE Conference, Seattle, WA, USA, 2–5 June 2014.

### 2.4. Effect of Shear on Crystallization in Mixed Polyolefins

McKenzie Coughlin *, Derek Huang, Anthony Kotula and Kalman Migler

National Institute of Standards and Technology
* Correspondence: mckenzie.coughlin@nist.gov

**Abstract:** Polyolefins, such as polyethylene (PE) and polypropylene (PP), are among the most abundant single-use plastics and contribute the largest share of the plastics waste stream. However, these polyolefins are typically mixed when they enter the waste stream, and have low recycling rates due to separation difficulties. Blends of PE and PP tend to have poor mechanical properties due to their thermodynamic immiscibility and the resulting micro-separated domains that crystallize in a cascading fashion upon cooling. The crystallization behavior of these mixed plastics during processing is critical to the overall mechanical properties, yet is poorly understood. In this work, we study the crystallization kinetics and rheological properties of mixtures of high-density PE and isotactic PP using a combination of rheo-Raman microscopy, electron microscopy, and differential scanning calorimetry. In particular, we examine the effects of shear, composition, and blend morphology on the resulting semicrystalline microstructure and the kinetics of flow-induced crystallization in each phase. We find that there is a composition dependence to the flow-induced crystallization behavior of polypropylene, which we attribute to differing micro-flow fields between morphologies. Our results demonstrate the importance of rheology and processing on the overall properties of mixed crystallizing recyclates. 

### 2.5. Very-Long-Time Crystallization in Ethylene–Methacrylic Acid Ionomers

Brian Grady *

University of Oklahoma
* Correspondence: bpgrady@ou.edu

**Abstract:** Papers by our group [1] and another group [2] explored the crystallization of random copolymers after room-temperature annealing of ethylene and methacrylic acid. Both papers reached similar conclusions regarding the nature of the crystallization process: primary crystals were either unchanged or changed very little after their initial formation, while secondary crystallization occurred over much longer time scales after annealing at room temperature. The current work confirms these general conclusions for four different cations (Zn^2+^, Na^+^, Li^+^ and Mg^2+^), with the latter two not studied in either of the previous studies. Even after 6 years of annealing at room temperature, the crystallization rate is constant with the logarithmic time, i.e., the material continues to crystallize. Secondary crystals of the acid copolymer thicken with time as well, and if the material crystallizes with time the rate is very slow.

**References**

1. Rui, Y.; Grady, B.P. Long-time crystallization kinetics in zinc-neutralized ethylene-methacrylic acid ionomers. *Thermochim. Acta* **2013**, *565*, 183–193.
2. Spencer, M.W.; Wetzel, M.D.; Troeltzsch, C.; Paul, D.R. Effects of acid neutralization on the properties of K^+^ and Na^+^ poly(ethylene-co-methacrylic acid) ionomers. *Polymer* **2012**, *53*, 569–580.

### 2.6. Fast Scanning Calorimetry of Polymers

Mark Weber * and Natalie Stingelin

Georgia Institute of Technology
* Correspondence: mweber47@gatech.edu

**Abstract:** Thermal characterization of polymers has provided insight into the diverse and frequently multiphase behavior of these macromolecular materials since the first synthetic efforts created them. Through thermal analysis, it has been revealed that polymers can, in the solid state, take on both ordered and disordered phases simultaneously. The degree to which these domains are present is highly dependent on both the processing and the thermal history. In particular, differential scanning calorimetry is used to measure phase transitions through changes in heat capacity or latent heat. Generally, ordered polymer phases melt with latent heats that are easily detectable when scanning at conventional rates on the order of tens to hundreds of degrees Celsius per minute. Polymer phase behavior, however, is often more subtle, and can exhibit multiple partially ordered phases, such as liquid crystallinity. The associated changes in the heat capacity and latent heat of these partially ordered phases are likewise subtle, and can be easily obscured. Because the measured heat flow is proportional to the scanning rate, scanning at increased rates can serve to enhance otherwise undetectable signals. Recent technological advancements have enabled fast differential scanning calorimetry which is capable of operating at scanning rates of several thousands of degrees Celsius per second. Herein, we survey the diverse obscured phase behaviors of a range of polymers from commodities, from polypropylene to semiconductors such as Poly[2,5-bis(3-hexadecylthiophen-2-yl)thieno[3,2-b]thiophene] (PBTTT). By virtue of fast scanning calorimetry, we observe subtle phase behaviors relevant to industrial production as well as to cutting-edge polymer research, laying the groundwork for future fast thermal characterization.

### 2.7. Selective Deuteration along a Polyethylene Chain: Measuring Differences in Conformation and Crystallization Segment-by-Segment

Aaron Burkey *, Anthony Kotula, Sara Orski and Kathryn Beers

National Institute of Standards and Technology
* Correspondence: aaron.burkey@nist.gov

**Abstract:** To improve the circularity and performance of polyolefin materials, recent innovations have enabled the synthesis of polyolefins with new structural features such as cleavable breakpoints, functional chain ends, and unique comonomers. As new polyolefin structures become synthetically accessible, fundamental understanding of the effects of structural features on polymer (re)processing and mechanical performance is increasingly important. While bulk material properties are readily measured through conventional thermal/mechanical techniques, selective measurement of local material properties near structural defects is a major characterization challenge. Here, we synthesized a series of polyethylenes with selectively deuterated segments and employed vibrational spectroscopy to evaluate crystallization and melting of chain segments near features of interest (e.g., end groups and mid-chain defects). Differences in conformational order were measured for different chain lengths and chain-end/backbone functionalities. Chain end functionality, in particular, was observed to strongly influence crystallinity and melting onset temperature of adjoining deuterated chain segments. Additionally, chain end crystallinity was observed to have different molar mass dependence than mid-chain crystallinity. The synthesis and spectroscopy techniques demonstrated here can be extended to a range of previously inaccessible deuterated polyethylene structures to provide direct insight into local crystallization behavior. With the ability to selectively measure conformation of specific chains or chain segments, we aim to enable more rational design of materials, blends, and (re)processing pathways to improve plastics circularity and material properties. 

### 2.8. Temperature Dependence for Poly(styrene) Depolymerization

Bob A. Howell *

Central Michigan University
* Correspondence: bob.a.howell@cmich.edu

**Abstract:** The development of polymeric materials in the mid-twentieth century has enhanced the quality of life in advanced societies around the world. However, the ever-increasing accumulation of polymeric waste, particularly plastics, and the depletion of resources has brought sustainability and recycling efforts to prominence. Depolymerization to generate monomers represents a preeminent goal for the recycling of polymer waste. Poly(styrene) is a prominent commercial polymer well-suited for recycling using this approach. However, progress has been hampered by a lack of an understanding of the details of the thermal degradation of poly(styrene). Thermal degradation of poly(styrene) has often been carried out at high temperature or using variable temperature techniques, conditions which mask important features of degradation processes. Most general-purpose poly(styrene) is produced under conditions which lead to polymerization termination by radical coupling. This places a head-to-head unit in the polymer mainchain. This unit is thermally labile and undergoes scission at modest temperature (280 °C), generating macroradicals which rapidly unzip to liberate styrene monomers. At this temperature, styrene monomers are effectively the only volatile species formed. In the presence of an effective hydrogen atom transfer agent, the macroradicals are rapidly trapped and the decomposition to form styrene monomers is suppressed. At higher temperatures (>300 °C), random chain scission may occur, forming a variety of products. If macroradicals are generated by other means, e.g., mechanical milling, depolymerization of poly(styrene) can be achieved at very low (ambient) temperatures.

### 2.9. Cooling Rate Dependence of Glass Transition in Semicrystalline Polymers

Joseph Menczel *

Thermal Measurements, LLC
* Correspondence: jmenczel@yahoo.com

**Abstract:** The glass transition of semicrystalline polymers is significantly different than the glass transition of amorphous materials. First, as was first found in the 1980s, there is a hysteresis peak when the sample cools slower than it reheats, leading to the apparent disappearance of the time dependence of the glass transition. Later, however, MT-DSC measurements recorded the hysteresis peak using the non-reversing signal, though with very small intensity. In addition, the missing ΔCp at the glass transition explained by the rigid amorphous fraction (RAF) can be used to explain certain phenomena during melting; polymers exhibiting cold crystallization have no RAF independent of the cooling rate when preparing (crystallizing) the sample. On the other hand, polymers with strong crystallization ability and high RAF (e.g., PBT) exhibit no or insignificant reorganization during melting. 

### 2.10. Rheological Hysteresis in Semicrystalline Polymers during Crystallization and Melting

Paul Roberts * and Anthony Kotula

National Institute of Standards and Technology
* Correspondence: paul.roberts@nist.gov

**Abstract:** Semicrystalline polymers such as polypropylene are known to exhibit history-dependent properties. This history dependence places additional emphasis on the polymer processing pathways used in manufacturing, as processing conditions must be designed with final product properties in mind. Here, we show that semicrystalline polymers do not melt in the same way that they crystallize, and that this difference has direct effects on the rheological properties of a material; it is possible for the same material at the same crystallinity to behave differently simply due to the direction in which the semicrystalline state was approached (i.e., heating or cooling). Using polypropylene as an example, we simultaneously measured small-amplitude rheological behavior and crystallinity during temperature sweeps using rheo-Raman spectroscopy. We observed that, for a given temperature, the rheological properties are dependent on whether the polymer is crystallizing or melting, that is, the polymer exhibits thermal–rheological hysteresis. Interestingly, and counterintuitively, we observed that a semicrystalline polymer can exhibit different rheological properties at the same crystallinity depending on whether the polymer is crystallizing or melting, that is, the polymer exhibits crystallinity–rheological hysteresis as well. 

### 2.11. Thermal Analysis and Rheometry Results from Ultra-Stable Glasses Challenge the Kauzmann Paradox

Dejie Kong ^1^, Amer El Banna ^1^, Yan Meng ^2^ and Gregory McKenna ^3,^*

1 Texas Tech University
2 Beijing University of Chemical Technology
3 North Carolina State University
* Correspondence: gbmckenn@ncsu.edu

**Abstract:** There is much theory based on the extrapolation of the thermodynamic and dynamic properties of crystallizing fluids to low temperatures where glass formation occurs. This results from an observation of a “paradox” in that the entropy of the liquid appears to go below that of the crystal, violating the Nernst third law of thermodynamics. This phenomenon is currently referred to as the Kauzmann Paradox. Associated with the Kauzmann temperature (T_K_) is the so-called Vogel–Fulcher–Tammann (VFT) temperature (T_VFT_), where the relaxation times or viscosity extrapolate to infinity. However, it is important to note that the theories that rely on T_K_ or T_VFT_ as evidence of an ideal glass transition are based on very large extrapolations of the temperature to below the glass transition temperature (Tg). Here, we use calorimetry and dynamic mechanical methods to show that it is possible to create (vapor-deposited amorphous Cytop) or discover (50 million year old amber) materials that have fictive temperatures (T_F_) that are below the T_VFT_, a situation that seems to contradict the idea that there is an ideal glass transition at T_K_ or T_VFT_. This is because the fictive temperature is notionally related to the “frozen-in” equilibrium state of the glass, suggesting that glass formation is a liquid state property that is not related to the Kauzmann paradox and possible crystallizable states in a material. In addition, we show evidence that the deep glassy state dynamic response in the nominal equilibrium condition where T_Test_ = T_F_, rather than being Arrhenius (or super-Arrhenius), is exponential in terms of temperature, i.e., k = A_0_exp (-E_a_/RT) over at least 22 orders of magnitude of relaxation time τ.

### 2.12. Crystallization of Conjugated Polymers: The Role of Interfaces, Molecular Attributes, and Thermal Processing

Lucia Fernandez Ballester *

University of Nebraska at Lincoln
* Correspondence: lucia.fernandez@unl.edu

**Abstract:** Semicrystalline polymers are extremely versatile materials with mechanical, optical, and diffusive properties dictated by their semicrystalline morphology. It is well known that crystallization of classical polymers is extremely sensitive to molecular characteristics and processing, meaning that even small changes can result in significantly different properties. For conjugated polymers, while charge transport critically depends on semicrystalline structure, the complex interplay between chain structure, processing, and crystallization remains elusive and hinders the development of a predictive model to attain optimal properties. Here, we investigate the role of interfaces, molecular attributes, and processing conditions on solvent-free crystallization of poly-3-heylthiophene (P3HT). In particular, we reveal a two-step crystallization process where the free surface of the film induces crystallization within the top 20 nm at temperatures 25 °C above bulk crystallization, resulting in high edge-on orientation and large birefringent structures at the air polymer interface [1]. We show that the role of molecular weight on P3HT crystallization strongly depends on regioregularity, and that self-seeding can be used to manipulate crystallization, though it is more effective in P3HT due to the presence of longer and more defective chains [2].

**References**

1. Hosseinabad, R.; Kuebler, J.; Fernandez-Ballester, L. Combined role of regioregularity and molecular weight on melt-crystallization and self-nucleation of poly(3-hexylthiophene). *Polymer* **2022**, *259*, 125341.
2. Kuebler, J.; Loosbrock, T.; Strzalka, J.; Fernandez-Ballester, L. Direct Observation of Two-Step, Stratified Crystallization and Morphology in Conjugated Polymer Thin Films. *Macromolecules* **2023**, *56*, 3083–3094.

### 2.13. Semiconducting Polymers: Nomenclature and Property Conundrums

Harald Ade *

North Caroline State University
* Correspondence: hwade@ncsu.edu

**Abstract:** Organic semiconducting polymer thin films are used in many organic electronic devices and have shown promising results in applications ranging from organic light-emitting diodes to solar cells and biosensors. The molecular packing of the polymers controls a number of optical and electronic properties, and the thermal and thermomechanical properties are critical for device stability in blends, processing strategies, and mechanical failure modes. Due to the molecular design, semi-conducing polymers exhibit a continuing range of paracrystalline disorder, yet at the same time they do not behave like classic paracrystalline materials. In fact, the classic structure and property paradigms and associated nomenclature do not seem to apply. Classic material design labels such as random coil, rigid rod, hairy rod, and bottlebrush seem to be delimiting structural characteristics, with semi-conducing polymers occupying the concept and parameter space in between these limits, yet with characteristics that appear to be not simply a linear combination of the characteristics found in the classical limits. We review the current understanding and the various conundrums. For example, paracrystallites in a semiconducting thin film are usually assumed to have a certain size and shape in three dimensions, which then can have a preferred edge-on or face-on orientation relative to the substrate with a random orientation in-plane. X-Ray analysis indicates though that most materials exhibit only two-dimensional paracrystalline platelet ordering, and might often resemble a layered glass. Thermomechanical characterization frequently indicates multiple relaxation transitions without the presence of a clear glass transition, yet with a high-temperature liquid-crystalline-like phase. Furthermore, the phase diagrams of semiconducting polymers with ‘small molecule’ acceptors such as fullerenes or modern non-fullerene acceptors frequently exhibit reentrant phase boundaries that reflect upper- and lower-critical solution temperature characteristics. The totality of information suggests that a new reference frame, if not a new paradigm, along with associated nomenclature, is required to facilitate understanding and communication about the order, molecular packing, texture, thermomechanical properties, and structure–property relations in semiconducting polymers.

### 2.14. Solidification Kinetics of Conjugated Polymers

Anush Singhal *, Mark Weber and Natalie Stingelin

Georgia Institute of Technology
* Correspondence: asinghal74@gatech.edu

**Abstract:** In recent years, synthetic efforts have led to radical improvement in the charge transport and energy harvesting properties of polymers designed for electronics. In particular, the properties of conjugated polymers, which are well suited for a variety of optoelectronic applications, have been enhanced. Due to their weak van der Waals interactions and conformation flexibility, conjugated polymers can be arranged into a multitude of morphologies. Because the relationship between material processing, structures, and properties are not well defined, optimal performance of conjugated polymers in optoelectronic applications has yet to be attained. Herein, we provide insight into the thermodynamic phase behavior of polymer semiconductors, making use of Poly(3-hexylthiophene) (P3HT) as a framework. By means of fast scanning thermal analysis, we describe the kinetics of nucleation, the growth of ordered phases, and the competition between existing phases. This work provides an understanding of solidification kinetics and its relation to the solid-state structure of P3HT. This insight can be extended to additional conjugated polymer systems, and can lead to the proposal of processing conditions to optimize the optoelectronic properties of semiconducting polymers.

### 2.15. Preservation of Crystallizability and Rapid Crystallization Kinetics of Blocky Brominated Poly(ether ketone ketone) Examined by Fast Scanning Calorimetry and Differential Scanning Calorimetry

Michelle Pomatto * and Robert B. Moore

Virginia Tech
* Correspondence: meeshp@vt.edu

**Abstract:** Block copolymers derived from engineering thermoplastics provide high chemical and thermal stability and good mechanical properties, as well as micro-phase separated morphologies that can significantly enhance material properties. Synthesis of block copolymers is complex, often involving inert conditions, highly controlled reaction sequencing, specialized initiators, and high-purity reagents. Our recent exploration of post-polymerization functionalization in the gel state led us to the discovery of suitable gelation conditions of poly(ether ketone ketone) (PEKK), allowing for functionalization in the gel state. Post-polymerization functionalization of PEKK in the gel state provides an efficient way to create blocky copolymers of PEKK, which may provide an enhanced alternative copolymer for improved water filtration, fuel cell membranes, or blend compatibilization. In this work, the phase separation of PEKK and a novel solvent is explored utilizing hot-stage microscopy and differential scanning calorimetry (DSC). Analysis of the phase-separated gel morphology is achieved by scanning electron microscopy. Additionally, the crystallization kinetics, crystallizability, and change in glass transition temperature of PEKK after post-polymerization functionalization in the blocky and random state has been achieved using fast scanning calorimetry (FSC) and DSC, while characterization of the blocky and random microstructure has been achieved using X-ray scattering.

### 2.16. Characterization of Fouling-Resistant Electrospun Nanofiber Membranes from Poly(vinylidene fluoride)/Polyampholyte Blends

Anuja Jayasekara *, Luca Mazzaferro, Ryan O’Hara, Ayse Asatekin and Peggy Cebe

Tufts University
* Correspondence: anuja.jayasekara@tufts.edu

**Abstract:** To improve the circularity and performance of polyolefin materials, recent innovations have enabled the synthesis of polyolefins with new structural features such as cleavable breakpoints, functional chain ends, and unique comonomers. As new polyolefin structures become synthetically accessible, fundamental understanding of the effects of structural features on polymer (re)processing and mechanical performance is increasingly important. While bulk material properties are readily measured through conventional thermal/mechanical techniques, selective measurement of local material properties near structural defects is a major characterization challenge. Here, we synthesized a series of polyethylenes with selectively deuterated segments and employed vibrational spectroscopy to evaluate crystallization and melting of chain segments near features of interest (e.g., end groups and mid-chain defects). Differences in conformational order were measured for different chain lengths and chain-end/backbone functionalities. Chain end functionality, in particular, was observed to strongly influence crystallinity and melting onset temperature of adjoining deuterated chain segments. Additionally, chain end crystallinity was observed to have different molar mass dependence than mid-chain crystallinity. The synthesis and spectroscopy techniques demonstrated here can be extended to a range of previously inaccessible deuterated polyethylene structures to provide direct insight into local crystallization behavior. With the ability to selectively measure conformation of specific chains or chain segments, we aim to enable more rational design of materials, blends, and (re)processing pathways to improve plastics circularity and material properties. 

### 2.17. Tuning Polymer Crystallization Pathway for Functional Materials

Christopher Li *

Drexel University
* Correspondence: chrisli@drexel.edu

**Abstract:** The polymer crystallization pathway is often sensitive to processing, external environments, and polymer chain architecture. This talk will discuss controlling the crystallization pathway to fabricate functional materials using soft confinement and varying the chain architecture. Crystallization at the liquid–liquid interface yields polymer crystals with unique chain folding and geometry. Introducing non-crystallizable moieties in molecular bottlebrushes significantly alters the morphology and structure of polymer crystals. The discussion will focus on shape-symmetry incommensurate crystals with broken translational symmetry. We emphasize that not only can tuning the crystallization pathway shed light on crystallization mechanisms, it provides a promising way to fabricate functional materials.

### 2.18. Leveraging Thermal Characterization to Optimize Materials and Process Development for Industrial Adhesives And Films

Alexander Bourque *

3M
* Correspondence: ajulesbourque@gmail.com

**Abstract:** Designing new materials for industrial applications is a complex task requiring the support of chemists, process engineers, application experts, and many more. At 3M, our collaborative teams use an abundance of materials characterization data to make critical decisions at all stages of product development. For polymeric materials, thermal characterization is an essential tool to probe thermo-responsive behavior and other temperature-activated processes. This talk will focus on two topics: (1) quantifying the cure state in a thermally activated curable adhesive to integrate into predictive models for product scale-up, and (2) optimizing processing conditions for bio-degradable polymer films using DSC. Through these examples, I will highlight the importance of thermal analysis to build fundamental materials understanding and how it can be effectively leveraged during industrial process scale-up.

### 2.19. Thermal Analysis in Defining the Structural Characteristics of Starch Materials

Janis Matisons *

Intertape Polymer Group
* Correspondence: jgmatisons@gmail.com

**Abstract:** Thermal analysis techniques have gained prominence in food research over the last two decades. Sensitive instrumentation developed for thermo-mechanical analysis (TMA) and differential scanning calorimetry (DSC) has allowed application of thermal analysis to the study of heat related phenomena in foods, particularly in the study of starch.

Phase transitions are extremely important both when using starch in manufacturing adhesives and in food processing. The melting and glass transition temperatures are the most important parameters that characterize physical properties of starch polymers over a wide temperature range. These transitions define and explain differences in the physical properties of starches. DSC provides valuable insights into the order–disorder transition of starch granules in water. Starch has a complex structure, and upon processing a different and potentially equally complex structure results. Despite the extensive use of DSC in starch analysis today, there are thermodynamic equilibrium issues that arise with respect to the large heating or cooling rates, as every system that undergoes a phase transition has its own internal relaxation time. This discussion will include what thermal analysis can teach us about this biopolymer.

### 2.20. Ultrasound-Regulated Protein-Based Films and Nanofibers for Biomedical Applications

Xiao Hu ^1,^* and Fang Wang ^2^

1 Rowan University
2 Nanjing Normal University
* Correspondence: hu@rowan.edu

**Abstract:** Ultrasound-assisted protein-based materials, both film and nanofiber, have been developed recently, allowing the structure of biocompatible and green protein materials to be physically manipulated in an efficient, repeatable, chemical-free, non-contact, and highly controllable manner. This technology produces a cavitation effect that promotes the generation of free radicals, the fracture of chemical chain segments, and a rapid change in morphology. With its high efficiency, cleanliness, and reusability applications, ultrasound has a vast range of opportunities within the field of natural polymer-based materials.

Our studies demonstrate that ultrasound can regulate protein structure at the solution assembly stage to obtain the desired properties of protein-based materials, and that these properties can be fine-tuned by altering variables. Ultrasound-regulated silk film materials showed higher thermal stability, better biocompatibility and breathability, and favorable mechanical strength and flexibility. It was possible to tune the enzymatic degradation rate and biological response (cell growth and proliferation) of silk protein materials by changing ultrasound parameters. Additionally, ultrasound-assisted air-jet spinning of silk–soy protein nanofibrous materials were fabricated with tunable properties, with the high biocompatibility providing a wide range of applications in wound dressings and drug-carrying systems. Various techniques were utilized to characterize the ultrasound-assisted protein-based materials, including Fourier transform infrared spectroscopy (FTIR), X-Ray diffraction (XRD), scanning electron microscopy (SEM), differential scanning calorimetry (DSC), thermogravimetric (TG) analysis, dynamic mechanical analysis (DMA), gas permeability, water contact angle measurements, enzymatic degradation, and cytotoxicity assays. These findings demonstrate the potential of ultrasound-assisted protein-based materials for sustainable development and improved healthcare outcomes.

### 2.21. Influence of Polymer Architecture on Catalytic Deconstruction of Polyolefins

Alex Balzer *

University of Delaware
* Correspondence: ahbalzer@udel.edu

**Abstract:** Plastics are an indispensable class of materials found in countless consumer products; yet, their growing use and limited recycling strategies have led to a rapid accumulation of waste plastics and many negative environmental impacts. Thus, efforts to improve end-of-life strategies to address plastics pollution are expanding. Polyolefins, which comprise over half of new plastics production, can be chemically recycled into valuable carbon feedstocks such as oils and lubricants. This deconstruction process is not trivial, and requires fundamental understanding of the polymer physical properties affected by molecular weight distributions and architectures, which vary widely across polyolefin feedstocks. We utilize thermal fractionation via self-seeding and annealing to separate polymer crystals by their methylene sequence lengths in order to determine the branching architecture of polyethylenes after chemical deconstruction. By combining thermal analysis with gel permeation chromatography, several reaction pathways found in hydrocracking, hydrogenolysis, and pyrolysis, such as isomerization and chain scission, can be tracked. The methylene sequence lengths and distributions of the neat polymer influence the deconstruction pathway and kinetics. By identifying trends in molecular weight distributions and branching architecture, our combined technique allows for further insight into other polymer physical processes that affect deconstruction, including adsorption and transport.

### 2.22. Thermal Transitions in Conjugated Polymers: Unraveling the Multiple Relaxation Signatures

Brendan O’Connor *

North Carolina State University
* Correspondence: btoconno@ncsu.edu

**Abstract:** Conjugated polymers have unique physical properties imparted by their relatively stiff donor–acceptor backbone and soft hairy side chains. The complex and large monomer structure can give rise to complex thermomechanical behavior that is difficult to characterize. Yet, the thermal relaxations have important implications for film processing and the resulting microstructure and mechanical behavior. In this talk, we report on the thermal relaxations observed in a set of systematically varied conjugated polymers (based on the PBnDT-FTAZ family) using dynamic mechanical analysis (DMA), as well as how the relaxation temperatures vary with subtle changes to the molecular structure of the polymer. We show that there are local thermal transitions to those that are more cooperative due to side-chain relaxations, including aggregate relaxation. Furthermore, we show the direct connection between the observed thermal relaxations and molecular packing of various conjugated polymers using in situ GIWAXS and in situ UV-visible light spectroscopy. We show how certain thermal transitions have features that are consistent with liquid crystalline behavior, but at the same time do not conform fully to liquid crystalline behavior. In addition, through this in situ characterization, we comment on the limitations of packing order in donor–acceptor conjugated polymers. Finally, we discuss how the various relaxations impact the stress–strain behavior of the film and how this information can be used to guide stretchable polymer semiconductor design.

### 2.23. Deciphering the Difference between Flexible Chain and Hairy Rod Polymer Semiconductors

Natalie Stingelin *

Georgia Institute of Technology
* Correspondence: natalie.stingelin@mse.gatech.edu

**Abstract:** Organic electronic materials possess unique opto-electronic and processing properties that provide broad opportunities for use in light-emitting diodes, solar-energy harvesting systems, to next-generation sensors and neuromophic computing components. These technologies have been continually improving over the past decades, aided by advancements in materials chemistry and processing innovation. State-of-the-art polymer semiconductors typically have an electron donor-acceptor (D-A) backbone structure with a number of fused ring moieties, and complex aliphatic or, e.g., ethylene-oxide side chains that decorate the backbone to provide solubility. It is important to recognize that the physical properties of the side chains are substantially different from the backbone, constituting an amphiphilic-like characteristics transverse to the backbone reminiscent of phospholipid and surfactant molecular analogs, and simple classical descriptions of amorphous vs. semicrystalline structure no longer apply. Here, we discuss how the FSC technique can be used for the identification of thermodynamic transitions of next generation D-A polymers commonly used in the organic solar cell area to obtain important structural information of this new class of materials and, in turn, establish processing guidelines towards materials of specific optical or electrical characteristics, and improved materials design for organic optoelectronic devices. 

## 3. Session: Energetic Materials and Thermal Hazards

### 3.1. Thermal Runaway in Nitric Acid-Soaked Organic Kitty Litter

Michael Hobbs *

Sandia National Laboratory
* Correspondence: mlhobbs@sandia.gov

**Abstract:** Precise wording is important in every field of study, including operational procedures. Confusion around the wording “organic” and “inorganic” may have contributed to an accidental substitution of an organic kitty litter for an inorganic adsorbent used to prepare nuclear waste for disposal at the Waste Isolation Pilot Plant (WIPP) [1]. The adsorbent was used to prevent liquids such as nitric acid from causing additional issues such as corrosion within the waste drums. However, combination of organic material with nitric acid can cause heat- and gas-generating reactions in which heat is generated faster than it can be dissipated, resulting in thermal runaway rapid pressurization and confinement breach.

On 14 February 2014, just before midnight, radioactive waste within a 55- gallon steel drum (designated 68660) thermally ignited 655 m (2150 ft) underground in a salt formation that is part of a nuclear waste repository located near Carlsbad, New Mexico, USA. The waste drum contained nitric acid-soaked organic kitty litter mixed with various metal nitrate hydrates, acid neutralizer (triethanolamine), rubber, plastic, a tungsten-lined glove, and transuranic elements (americium-241 and plutonium-239) [2]. Prior to this event, 143 drums with similar reactive contents had been placed in the repository without incident.

Following the breach of drum 68660, a technical assessment team from various laboratories reviewed historical drum content data, performed numerous experiments, and attempted to reconstruct the event using computational models. However, no conclusive evidence of the cause of the thermal runaway within drum 68660 was identified. Recently, we were able to simulate thermal ignition in drum 68660 using a pressure-dependent waste decomposition model [1] calibrated with data from full- scale drum experiments [3] and validated with experiments from multiple laboratories [4]. We conclude that a restricted drum vent could have led to the thermal runaway reaction in drum 68660.

**References**

1. Hobbs, M.L.; Britt, P.F.; Hobbs, D.T.; Kaneshige, M.J.; Minette, M.; Mintz, J.; Pennebaker, F.M.; Parker, G.R., Jr.; Pierce, R.; Rosenberg, D.M.; et al. Thermal runaway of nitric acid-soaked kitty litter in transuranic waste. *Process Saf. Environ. Prot.* **2022**, *167*, 543–549. https://doi.org/10.1016/j.psep.2022.09.047.
2. Wilson, D.L.; Baker, M.L.; Hart, B.R.; Marra, J.E.; Schwantes, J.M.; Shoemaker, P.E. Waste isolation pilot plant technical assessment team report. *Savannah River Natl. Lab. Rep. SRNL* **2014**. https://doi.org/10.2172/1764818.
3. Parker, G.R.; Holmes, M.D.; Heatwole, E.M.; Dickson, P.; Leonard, P.; Leibman, C.P. The thermolytic response of a surrogate RNS waste mixture at the drum scale. *Los Alamos Natl. Lab. Rep* LA-UR-15-29229, **2016**.
4. Hobbs, M.L.; Britt, P.F.; Hobbs, D.T.; Kaneshige, M.J.; Minette, M.; Mintz, J.; Pennebaker, F.M.; Parker, G.R., Jr.; Pierce, R.; Rosenberg, D.M.; et al. Modeling delayed thermal runaway in nitric acid-soaked cat mixed with radioactive waste. *J. Therm. Anal. Calorim.* **2023, submitted**.

### 3.2. Material Relocation Study of Pellet–Cladding Interaction in CFR600 Design

Yann Peng *

China Institute of Atomic Energy
* Correspondence: pengyan@ciae.ac.cn

**Abstract:** Thorough study of material relocation of pellet–cladding interaction is necessary in CFR600 design [1]. The key to material relocation research is determination of the melting point temperature of the fuel pellet and cladding during PCI. Typically, melting belongs to the first-order phase transformation.

In order to obtain the melting temperature of PCI it is better to depend on the phase diagrams, which provide a graphic representation in space of the domains of stability of various phases that an alloy can exhibit at equilibrium in the solid, liquid, and gas states. When obtaining these metallurgical phase diagrams, it is known that under certain temperature and pressure conditions the products of phase transfer in the melting phase can be confirmed through the value of the Gibbs energies [2]; moreover, the phase with the lowest Gibbs energies can exist stably. In fact, while the crystal is melting, the crystal Gibbs energies before melting and after melting are the same. Hence, at a fixed pressure the curves of the solid phase and liquid phase changes with the temperature intersect, and the temperature at the intersection point is the melting temperature of the materials. Based on the chemical potential and Gibbs energy distributions, the metallurgical phase diagram determined by DSC/DTA can provide the necessary information on the melting temperature.

Therefore, this material relocation study (test and simulation) can yield a better understanding of the phenomena involved in this interaction between the fuel pellet and the cladding in CFR600 design [3].

When cladding temperatures exceed the 15-15Ti melting temperature, this results in local cladding material relocation and fuel dissolution. In particular, the ballooning of the fuel rods due to relocation behavior contributes to an early increase in the bundle flow resistance which is delayed until the onset of melt relocation.

In the chemical interaction of PCI, when the temperature of the interface surpasses the melting temperature of the low-melting phase, the interpenetration of the liquid phase can penetrate into the cladding. Indeed, some of these relocated materials can be inferred from abrupt and coincident changes in the response of the thermocouples at different axial levels and among different bundle components. Based on these coincidental changes in the response of the thermocouples, it is possible to test the origin of relocated materials, the relocation distance, the effect of relocation on local temperature, and the velocity at which the molten material was relocated.

In addition, fission-enhanced melting can occur. A higher fission-to-chemical power ratio leads to the formation of large amounts of melting and the attainment of greater superheats, both of which promote the formation of large melts that subsequently form cohesive blockages. It is highlighted that the effects of different fission-to-chemical power ratios cannot be accurately quantified without an accurate relocation model that can identify the processes of penetration and diffusion.

**References**

1. Siefken, L.J.; Coryell, E.W.; Harvego, E.A.; Hohorst, J.K. Modeling of Reactor Core and Vessel Behavior During Severe Accidents. *U. S. Nuclear Regulatory Commission Code Manual 6150, Volume 2, Rev. 2 INEL-96/0422* **2001**.
2. Clark, D.L. Geeson, D.A.; Hanrahan, R.J. *Plutonium Handbook*; American Nuclear Society: La Grange Park, IL, USA, 2019.
3. Wang, Z.; Cao, X.W. Preliminary Thermal-Hydraulic Phenomena Investigation during Total Instantaneous Blockage Accident for CEFR. *Nucl. Eng. Des.* **2007**, *237*, 1550–1559.

### 3.3. Using Differential Scanning Calorimetry for Chemical Compatibility Analysis of High Thermal Stability Explosives

John Rosener *

Lawrence Livermore National Laboratory
* Correspondence: rosener1@llnl.gov

**Abstract:** Chemical compatibility is a topic of concern for both commercial and government entities. If two substances are not chemically compatible, deleterious reactions such as excessive heat evolution, noxious byproduct generation, or worse can occur. When it comes to chemical compatibility of energetic/high explosive materials, safe handling aspects are the foremost concern. If an energetic material is incompatible with another substance, the energetic material could change and become more sensitive to detonation stimuli or generate enough heat to encourage runaway reactions. Either outcome could result in unintentional detonation and could put peoples’ lives at risk. A secondary concern for the chemical compatibility of energetics is degradation of performance. If an energetic is in intimate contact with an incompatible material within an assembled device (e.g., a military munition), the energetic material could degrade to such an extent that the device can no longer perform as designed.

Energetic chemical compatibility can be measured using a variety of techniques. The two most common methods employed at Lawrence Livermore National Laboratory (LLNL) are the Chemical Reactivity Test (CRT) and Differential Scanning Calorimetry (DSC). CRT is a thermal aging test in which a 1:1 mixture of energetic and alien material is loaded into an airtight vessel. The vessel is isothermally aged for a certain period of time. After the aging period is complete, the headspace within the vessel is sampled and analyzed via gas chromatography (GC). The GC is calibrated to measure the volume of specific gases which are known to be products of energetic molecule decomposition. For DSC, a 1:1 mixture of energetic and alien materials is loaded into a DSC pan and the mixture is heated from room temp (RT) to 500 °C at a constant rate. The exothermic peaks from the mixture and a pure energetic sample are measured and compared in order to determine whether there are significant shifts in peak shape or exotherm onset temperature.

DSC is an older technique than CRT, and has been used for a longer period. Issues have arisen when using DSC to evaluate the chemical compatibility of energetics with higher thermal stability. Newer energetic molecules and formulations possess higher exothermic onset temperatures than legacy materials. This higher thermal stability is by design, intended to make new energetic materials safer to handle and store. However, when these new materials are tested with common alien materials, such as polymers and adhesives, the difference in exothermic onset temperatures can be very stark. Depending on the type of polymer or adhesive used, the difference in exothermic onset temperature between a pure energetic and a mixture could differ by 100 °C or more! These large differences in onset temperature have led to confusing results and have complicated discussions regarding material safety and handling requirements. This talk will address difficulties with interpretation of DSC chemical compatibility data and highlight workarounds that can help to support or refute confusing results.

### 3.4. Studies of Ferrocene-Based Ionic Liquids Applied as Burning Rate Catalysts for Composite Solid Propellants through Thermal Methods

Cesar Morales *

Universidad Bernardo O’Higgins
* Correspondence: camoralv@uc.cl

**Abstract:** Rocket technology investigation is currently garnering a lot of interest because of its potential applications, particularly in the aerospace and defense industries. This is a crucial step on our route to expand human exploration into space. Burn Rate (BR) Catalyst studies on thermal degradation of primary oxidizing agents for the composite solid propellant ammonium perchlorate (AP) is fundamental for application in rocket motors to improve thrust and acceleration in relation to the hot gases inside the combustion chamber that produce propulsion.

Therefore, the impact on the thermal decomposition of AP is often used to evaluate the combustion effect of a BR catalyst candidate on the combustion behavior of composite solid propellants. This work aims to describe the catalytic effect of Ferrocene-based ionic liquids by thermogravimetry and Differential Scanning Calorimetry (DSC) techniques, as this effect can significantly decrease the decomposition temperature of AP, resulting in improved performance of the composite solid propellant and increased energy release.

### 3.5. The Reaction Kinetic Handling of Complex Reaction Behaviour in CTPB-MAPO Solid Rocket Propellants

Manfred Bohn *

Fraunhofer ICT
* Correspondence: Manfred.Bohn@ict.fraunhofer.de

**Abstract:** Generally, the reactions in energetic materials are complex, and are not deconvolutable with typical thermoanalytical evaluations. High-temperature analysis of solid composite rocket propellants ( CRPs) does not provide the essential reaction data necessary for predicting ageing and service life at in-service temperatures, as the decomposition of AP and the connected reactions are the main effects seen with DSC and TGA. Service life of assets using CRP is related to the safety and functional reliability of the system, two features that are often controlled by changes in mechanical properties caused by entirely different reactions than decomposition of AP, such as binder oxidation or degradation.

Examples of a complex reaction behaviour include the curing of CTPB (carboxyl-terminated polybutadiene) with aziridine-type curing agents such as MAPO (tris[1-(2-methylaziridinyl)] phosphine oxide). MAPO shows a special behavior; in addition to the main curing reaction, it shows splitting of the binder network by N-P bond breakage inside its own chemical frame. CTPB binders have interesting properties in that the bonding between solid particles and binder is supported by more polar molecular sites, contrary to HTPB binder. Therefore, such CTPB binder systems remain in applications with solid CRP. To handle this complex reaction behavior, suitable information must be available. One important property is the strength of the CRP, measured by tensile testing and establishing the stress–strain curve. The strength is often used as an index of failure, and is related to a failure criterion used to assess whether a load will induce damage in the structural entity of the propellant. The Young’s moduli are extracted from the stress–strain curves and used in kinetic modelling. For this, several composed reaction kinetic models have to be established and applied to the data. This procedure is shown and discussed.

### 3.6. Determining Safe Propellant Quantities in Locally Operated Manufacturing Environments

Frederick Paquet ^1,^* and Mario Paquet ^2^

1 Frederick Paquet Scientific Consulting
2 General Dynamics—Ordnance and Tactical Systems—Valleyfield Canada
* Correspondence: frederick.paquet@usainteanne.ca

**Abstract:** A current research trend in energetic materials safety is devoted to exploring the various behaviors of 1.3 materials [1,2]. These materials, often solid propellants, are known to exhibit behaviors ranging from common organic substances fires to detonations [2]. How a combustion event unfolds depends on the nature of the burning material and its configuration. For example, one can compare a rocket motor fire in an open area stack and the ignition of small arms propellant in a semi-enclosed glazing barrel. Technological advances have helped in switching from manufacturing methods involving exposed personnel to remote processing. Nevertheless, it is not always possible or practical to eliminate the exposure to 1.3 products. Therefore, it is necessary to design safe limits and good conduct guidelines by which these activities should occur. While such standards exist for military ammunitions and explosives, they are lacking in manufacturing environments.

This study applies previously derived results to determine safe exposure quantities for 1.3 propellant manufacturing environments. Such quantities are paramount in the design of a proper basis of safety for these materials. This determination is obtained by considering the radiant heat flux level during events [3], safe thermal doses [4], fire plume dimensions [2], pressure generation, and venting conditions [5]. In additions, general egress rules must be applied to estimate a safe available time during events. This helps to set the basic framework on which to design new installations. Obviously, specific cases may require specialized treatment; however, the methodology would remain the same. Hence, this approach yields guidelines for clearly safe, clearly unsafe, and ambiguous situations. In the organization responsible for this research, this is in the process of becoming the basis of safety for dry propellants. Ultimately, such guidelines would become available to the industry in the form of standards.

**References**

1. Paquet, F. The Fire Safety of Granular Propellant Handling Facilities. Thesis, 2017.
2. Covino, J.; Romo, C.P.; Phillips, J.W.; Atwood, A.I.; Boggs, T.L. Combustion Behavior and Quantity Distance Siting. In Proceedings of the International Explosive Safety Symposium, 2018; National Defense Industrial Association: San Diego, CA, USA, 2018.
3. Paquet, F.; Ng, H.D.; Paquet, M.; Cottin, F.; Groulx, D. Modeling propellant fires radiant heat flux. *J. Energetic Mater.* **2019**, *37*, 110–124.
4. US Department of Defense. Unified Facilities Criteria—Structures to Resist the Effects of Accidental Explosions. *UFC 3-340-01*. 2008.
5. Paquet, F.; Ng, H.D.; Paquet, M.A general pressure generation model for granular propellant fires. In Proceedings of the International Explosive Safety Symposium, 2018; National Defense Industrial Association: San Diego, CA, USA, 2018.

### 3.7. Tracking the Explosive Signature

Jimmie Oxley ^1,^*, James Smith ^1^, Thomas Lenehan ^1^ and Brian Giebel ^2^

University of Rhode Island
City University New York
* Correspondence: joxley@chm.uri.edu

**Abstract:** Several studies have investigated the fate and transformation of energetic materials when disposed of in soil. Here, we ask how being spalled from an explosive under tens of gigapascals of pressure and a temperature in the thousands of degrees changes the explosive signature, and specifically, the isotopic signature. Our approach is the following: (1) characterization of the intact explosive; (2) subjecting the explosive to detonation over soil; (3) quantifying the amount of the original explosive captured; (4) identifying, where possible, the detonation products; (5) using mass spectrometry (MS) to determine the C and N isotopic ratios (IRMS) in the original explosive before and after it was subjected to detonation; (6) determining the changes in isotope ratios as the explosive is allowed to sit in soil. Studies were performed on 2,4-dinitroanisole (DNAN) due to its tendency to leave residue.

### 3.8. Why Dispersion Pressure Has Dominant Effects on the Minimum Ignition Temperature of Combustible Dust Clouds: Examples of Australian Coal Dust, Corn Starch, and Lycopodium Powder

Gan-Syue Guo and Chi-Min Shu *

Department of Safety, Health, and Environmental Engineering
National Yunlin University of Science and Technology
* Correspondence: shucm@yuntech.edu.tw

**Abstract:** The minimum ignition temperature of a combustible dust cloud (MITC) is a crucial parameter for dust hazards; the analysis method follows IEC 80079-20-2. However, this standard does not include a detailed definition and description of the choice dispersion pressure. This study employed a Godbert–Greenwald furnace and high-speed camera to investigate the examples of Australian coal dust, corn starch, and lycopodium powder and reckon the flame propagation speed. The flammable limit concentration (FLC) of the dust was computed and compared with the minimum explosive concentration (MEC). The results showed that the MITC of lycopodium and corn starch increased with decreasing dispersion pressure, while there was no salient difference in the coal dust. A dispersion pressure of 0.5 barg resulted in dust with a higher flame propagation velocity. FLC and MEC varied depending on the ignition source and the presence or absence of dust dispersers. It is recommended to set the dispersion pressure above 0.5 barg in order to ensure correct thermal hazard parameters.

### 3.9. New Thermal Simulation Software for Chemical Reactions in Large Volumes

Elena Moukhina *

NETZSCH Geraetebau GmbH
* Correspondence: elena.moukhina@netzsch.com

**Abstract:** In real tasks such as prediction of storage or transportation of highly energetic materials in the chemical industry, the temperature gradients in the reacting volume are significant and must be considered. For highly exothermal reactions, areas with higher temperature and faster reactions have more intensive heat production and self-heating. Such local areas can become hotspots for the beginning of runaway or thermal explosion. 

Although many FEM software systems can calculate the heat transfer, they tend to have problems when complex multi-step chemical reactions with thermal effects are present. Usually, such systems work well for model-free kinetics with single kinetic equation or for models with only one or two steps in which all kinetic parameters are known.

Here, we present our new Thermal Simulation Software which has no limitations for chemical processes. It is completely compatible with NETZSCH Kinetics Neo Software and is able to model both free and model-based approaches. For model-based approaches, there are no limitations on the number of individual reaction steps or the connections between them, including independent, competing, or consecutive ones.

The simulation software accepts all kinetic parameters directly from Kinetics Neo, then additionally uses temperature-dependent parameters such as density, thermal conductivity, and heat capacity. Each plane of the volume can contain its own material and thickness and its own surrounding material with a unique temperature profile. 

The software provides time-dependent and temperature-dependent results for the temperature and concentration of all reactants along with the reaction rate in both 2D and 3D views. Searching of the Self-Accelerating Decomposition Temperature (SADT) as well as simulation of adiabatic conditions and infinite heat transfer to the surroundings is available as well.

### 3.10. Components and Thermal Hazard Characteristics of Spark Fireworks and Cold Light Fireworks Tested by Simultaneous Thermogravimetric Analysis

Yia-Cha Ye * and Chi-Min Shu

Department of Safety, Health, and Environmental Engineering
National Yunlin University of Science and Technology
* Correspondence: in16882@gmail.com

**Abstract:** Among the nine categories of pyrotechnics regulated by the “Regulations on the Administration of Pyrotechnics”, the spark type is the third most imported normal pyrotechnic type in Taiwan. It is widely used in festivals and public events. Professional stage pyrotechnics (cold light fireworks) and normal spark light fireworks have the same discharge pattern. Normal spark light fireworks make a large amount of smoke and have high temperature on discharges, with a composition of potassium perchlorate and Al-Mg metal powder. On the contrary, the main component of cold light fireworks is nitrocellulose. When combined with titanium metal powder, they have the characteristics of a low ignition point and lower temperatures, and do not generate dense smoke.

In terms of discharge temperature, cold light fireworks are safer than normal spark light fireworks; the average temperature generated by the former was 201.49 °C, while for the latter it was 503.38 °C. The former type is relatively more environmentally friendly due to the lack of dense smoke. However, it should be noted that the presence of “nitrocellulose” in fireworks grants them nitrating properties and a higher energy content than normal spark light fireworks, which have an average enthalpy of 567.42 J g^−1^, compared to 2389.64 J g^−1^ for fireworks containing nitrocellulose. This poses potential thermal hazards, and both types of fireworks have different apparent activation energies as measured by the ASTM E698 method. The former requires less energy and is more reactive than the latter, indicating the importance of carefully managing the amount of nitrocellulose during production and storage.

This study used commercially available normal spark light fireworks and cold light fireworks. These fireworks produced a relatively simple spark eruption, and the gunpowder was sampled directly without grinding or additional sieving. To ensure the accuracy of the experimental results, laterite fixtures on the cylindrical launch port and lime fixtures on the base were excluded as much as possible to avoid contamination of the sampling samples, then observation of the microstructure was conducted through field emission scanning electron microscopy (FE-SEM)/energy-dispersive X-Ray spectroscopy (EDS), thermal analysis using a laser particle sizer, and simultaneous thermogravimetric analysis (STA) while using advanced thermodynamic analysis software to compare and discuss the thermal hazard characteristics of the two types of fireworks.

### 3.11. Thermal Decomposition of Fluorinated Polymers in Plasticized Explosives

Athina Kominia, James Smith and Jimmie Oxley *

University of Rhode Island
* Correspondence: joxley@chm.uri.edu

**Abstract:** Because military explosives require long-term stability, fluoropolymers have been used as a binder or plasticizer in several explosive formulations and munitions. However, it is inevitable that everything becomes unserviceable and obsolete eventually. Open burn and open detonation (OB/OD) and incineration are usually the disposal methods of choice. In recent years, concerns have risen regarding the effect on human health and development caused by the high concentration of these indestructible plastics and their byproducts. They have been found in the air, in food and drink, and in the human body. Here, we examine the potential environmental release of fluorinated species from such thermal treatment.

### 3.12. The Process Safety Vessel Used in Calorimetry Studies of an Energetic Material

**Shanti Singh, Hope Dettweiler, Garrison Condran**

Canadian Explosives Research Laboratory
* Correspondence: shanti.singh@canada.ca

**Abstract:** Experiments using a process safety vessel (PSV) were performed to assess thermochemical data for use in risk analyses. The PSV was housed in the sample well of a heatflow calorimeter, and contained two concentric quartz tubes, with each containing a chemical of interest. The vessel design allows for remotely breaking the inner tube to initiate contact between the two chemicals. PSV calorimetry data are presented for two systems; ammonium nitrate/sodium nitrite, and biochar/sodium nitrate.

© His Majesty the King in Right of Canada, as represented by the Minister of Natural Resources, 20234.13. Continuous monitoring of the shelf life of energetic materials. Application in novel kinetic analysis of the information supplied by data loggers.

### 3.13. Continuous Monitoring of the Shelf Life of Energetic Materials. Application in Novel Kinetic Analysis of the Information Supplied by Data Loggers

Bertrand Roduit ^1,^*, Patrick Folly ^2^, Alexandre Sarbach ^2^ and Richard Baltensperger ^3^

1 AKTS SA
2 armasuisse
3 University of Applied Sciences of Western Switzerland
* Correspondence: b.roduit@akts.com

**Abstract:** Most goods made by human hands are subject to decay, the rate of which determines their shelf life (SL). In general terms, SL can be defined as a finite period after production during which the product retains a required level of quality under well-defined storage conditions.

To be considered applicable and/or safe, the products:

(i) Must fulfil the properties set by the producers during their SL;

(ii) Their Self-Accelerating Decomposition Temperature (SADT) must meet the regulations set by the United Nations (UN) for the storage and transport of dangerous goods.

The SL of a product is directly related to the rate of its deterioration, which depends on external parameters, among which the temperature plays the most crucial role. Predicting when the acceptability limit of quality is reached requires knowledge of the kinetics of deterioration. This issue becomes even more critical in the last leg of the supply chain, when the temperature fluctuates during storage or transport. Therefore, the proper evaluation of the current state of the ageing degree of materials requires advanced kinetic tools which can continuously predict the rate of material deterioration at any time along with the temperature profile and localization during transport. In the present study, we propose a solution which applies an online system that receives the temperature (T) data supplied by T-data-loggers using Internet of Things (IoT) capabilities. By combining onboard GPS for continuous geo-location, a “Shelf-Life Monitoring System” has been developed to provide a global solution for remote 24/7 tracing, monitoring, and prediction of the degree of material degradation and thermal safety in any location and any environment. Due to the continuous monitoring of the reaction progress, the kinetic parameters allow the remaining shelf life of the materials to be under at any storage or transport conditions. The potential of the proposed approach is wide-ranging, including the pharmaceutical (vaccines, drugs), food, and chemical industries and safety applications for the surveillance of dangerous goods. The cost of implementing the Internet of Things (IoT) capabilities during transport and storage are covered by the savings flowing from reduced product waste and accident prevention.

## 4. Session: Thermophysical Properties

### 4.1. Preventing Pitfalls: Ensuring Material Compatibility in High-Temperature DSC Experiments

Jean-Philippe Harvey ^1,^*, Kentaro Oishi ^2^, Paul Lafaye ^3^ and Juan-Ricardo Castillo-Sanchez ^1^

1 Centre for Research in Computational Thermochemistry
2 CRCT—Polytechnique Montreal
3 Ecole Polytechnique de Montreal
* Correspondence: jean-philippe.harvey@polymtl.ca

**Abstract:** This study aims to explore the use of FactSage, a thermodynamic simulation software, in improving the efficiency and reliability of high-temperature Differential Scanning Calorimetry (DSC) analysis. Ensuring material compatibility in such experiments is a critical challenge that can significantly impact the validity of results and overall instrument health. Current approaches to assess material compatibility, including empirical knowledge, trial-and-error methods, and thermochemical databases, are often resource-intensive and can provide incomplete information. In this context, we propose the integration of FactSage simulations into the experimental design process as a more systematic and efficient solution. We demonstrate through a series of case studies how FactSage can accurately simulate material interactions, enabling researchers to predict potential compatibility issues before conducting actual experiments. We further explore the capability of FactSage to simulate Thermogravimetric (TG) and DSC signals, providing additional value in planning and interpreting thermal analysis experiments. Additionally, we provide practical guidelines on best practices for ensuring material compatibility in high-temperature DSC experiments and the effective integration of FactSage simulations into the experimental process. This research aims to serve as a valuable resource for researchers and practitioners in thermal analysis, assisting in avoiding common challenges and improving the overall quality of their high-temperature DSC studies.

### 4.2. Developing a New Set of Calibration Alloys for High-Temperature Thermal Analysis

Kil-Won Moon ^1,^*, Jiwon Park ^2^, Joo-Hee Kang ^2^, Chang-Seok Oh ^2^, Carelyn Campbell ^1^ and Ursula Kattner ^1^

1 National Institute of Standards and Technology
2 KIMS
* Correspondence: kil-won.moon@nist.gov

**Abstract:** The accuracy of high-temperature thermal analysis is often hampered by the lack of suitable calibration reference materials between the melting point of Au (1064 °C) and the melting of Ni (1455 °C). Additional uncertainty is associated with using Ni as a reference point due to the oxidation and vaporization of Ni at the melting point. This work is focused on developing new reference materials to serve as calibration standards for high-temperature thermal analysis. Five binary eutectic alloys have been developed that will reduce the uncertainty from greater than ±10 °C to less than ±2 °C and make the temperature calibration of commercial DSC/DTA within an interpolation range.

### 4.3. Thermophysical Properties of Fluids—Care, Collecting, and Connecting

Tina Adams * and Chris McFadden

The Lubrizol Corporation
* Correspondence: tina.adams@lubrizol.com

**Abstract:** Precise wording is important in every field of study, including operational procedures. Confusion in the wording “organic”; and “inorganic” may have contributed to an accidental substitution of an organic kitty litter for an inorganic adsorbent used to prepare nuclear waste for disposal at the Waste Isolation Pilot Plant (WIPP) [1]. The adsorbent was used to prevent liquids like nitric acid from causing additional issues such as corrosion within the waste drums. However, combination of organic material with nitric acid can cause heat- and gas-generating reactions in which heat is generated faster than it can be dissipated resulting in thermal runaway, rapid pressurization, and confinement breach.

### 4.4. Anisotropy of Thermophysical Properties in Extruded Polymers

Dale Hume * and David Landry

Thermtest Inc.
* Correspondence: dhume@thermtest.com

**Abstract:** The transient plane source (TPS) method can be used to simultaneously measure through-plane and in-plane thermal conductivities and thermal diffusivities if the volumetric heat capacity is known a priori. Four polymers—high density polyethylene (HDPE), polypropylene (PP), polystyrene (PS), and Nylon 66—are measured with the transient plane source method for their directional thermal properties. The measurements show significant anisotropy related to the direction of extrusion.

### 4.5. Thermal Diffusivity Test Study for Pellet–Cladding Interaction in CFR 600 Design

Yan Peng *

China Institute of Atomic Energy
* Correspondence: pengyan@ciae.ac.cn

**Abstract:** Pellet–Cladding Interaction (PCI) phenomena research is an important part in CFR600 design. In PCI study [1], the importance of the functions due to the boundary and interface concentrations and diffusion coefficients in analyzing the interdiffusion has to be highlighted. In order to determine this kind of diffusion bonding between the fuel pellet and the cladding and the changes with increasing burn-up, the metallurgical phases of both the fuel and cladding must be determined by Differential Scanning Calorimetry/Differential Thermal Analysis(DSC/DTA) [2,3]. Furthermore, the atomic interactive potential and the activation energy, which are the driving forces of thermal diffusion to control the PCI interface movement, should be tested as well.

In Fast Reactors, including CFR600, after the phase diagram information is available, the series work can be addressed, specifically, the information of the phase in a particular combination of temperature and composition, the proportion of the various phases, the heat treatments for optimizing structures and properties, the existing or avoided stable and meta-stable phases, the critical temperature at which other phases appear or disappear by solid-state transformation, and the effect of solute addition and variability from the nominal alloy composition on stability [4]. Obviously, the phase diagram is reliant on the basic laws of thermodynamics, which have a strong relationship with the changes in entropy and enthalpy. Both of these are functions of the temperature.

The diffusion coefficients of phase transitions and phase transformations are themselves changed due to the changes in chemical potential and the mole fractions. If the rate of reaction diffusion is controlled by the atomic diffusion rate (during the later period of reaction diffusion), then, the depth χ of reaction diffusion (the same as the interface movement rate) versus the time t will be χ = mt^1/2^; if it is controlled by the chemical reaction rate (during the beginning period of reaction diffusion), then the depth χ of reaction diffusion (the same as the interface movement rate) versus the time t will be χ = nt [5]. Furthermore, if the concentration of the interface is jumped (if a mixture region of the two phases exists), then their chemical potentials are equal in the phase balance conditions without any driving force of diffusion. Thus, the penetration texture of the binary alloy reaction diffusion does not have the mixture region of two phases.

Therefore, this paper elaborates the way in which thermal analysis measurements can be used to identify the activation energy and the interaction potentials of PCI and then to profile its metallurgical phase diagrams to confirm the corresponding thermal diffusion coefficients. Both of these can be published in the CFR600 design to greatly and effectively guarantee this reactor safety property from the point of setting criteria for controlling the penetration depth of PCI, similar to the evaluation criterion for the cladding penetration rate in NUREG-1368 for PRISM. The deepest penetration in the 1.0 h 1075 K test was 55 μm, which corresponds to a penetration rate of 1.5 × 10^−2^ μm per second.

**References**

1. Baker, R.B. Calibration of a Fuel to Cladding Gap Conductance Model for Fast Reactor Fuel Pins. *HEDL-TME 77-86 UC-79*. 1978.
2. Moukhina, E. Determination of Kinetic Mechanisms for Reactions Measured with Thermoanalytical Instruments. *J. Therm. Anal. Calorim*. **2012**, *109*, 1203–1214.
3. Boettinger, W.J.; Kattner, U.R.; Perepezko, J.H. DTA and Heat-flux DSC Measurements of Alloy Melting and Freezing. *National Institute of Standards and Technology, Special Publication 960-15*. 2006.
4. Clark, D.L.; Geeson, D.A.; Hanrahan, R.J. *Plutonium Handbook*; American Nuclear Society: La Grange Park, IL, USA, 2019.
5. Castleman, L.S. An Analytical Approach to the Diffusion Bonding Problem, *Nucl. Sci. Eng. ANS* **1958**, *4,* 209–226.

### 4.6. Comparative Analysis of Phase Transformations in Additively Manufactured vs. Conventionally Wrought Precipitation Hardening Martensitic Stainless Steels

Kil-Won Moon *, Saadi Habib and Fan Zhang

National Institute of Standards and Technology
* Correspondence: kil-won.moon@nist.gov

**Abstract:** This study focuses on the complex phase transformations in wrought and additively manufactured (AM) precipitation hardening martensitic stainless steels. We employ Differential Scanning Calorimetry (DSC) and a dilatometer for thermal analysis, shedding light on the phase transformation steps involving martensite, austenite, and precipitation. To validate and supplement these thermal measurements, we performed in situ, synchrotron-based, high-energy X-Ray scattering, and diffraction measurements, which provide structural insight into the nature of the phase transformations. A pivotal element of our investigation is the detailed comparison of the phase transformation pathways between AM and conventionally wrought alloys. This comparison is crucial, as AM can yield materials exhibiting different behaviors, even when the starting powder compositions align with the alloy composition specifications. Through this comprehensive analysis, we unveil the unique characteristics of phase transformations in AM material, thereby enhancing our understanding of their potential in AM contexts.

## 5. Session: Thermal Transport Measurements for Semiconductors and Electronic Device Materials

### 5.1. The Role of Interfaces in Thermal Management Strategies For Ultrawide Bandgap Electronics

Samuel Graham ^1,^*, Henry Aller ^1^, Patrick Hopkins ^2^ and Asif Khan ^3^

1 University of Maryland
2 University of Virginia
3 University of South Carolina
* Correspondence: samuelg@umd.edu

**Abstract:** Ultrawide bandgap semiconductors made from high Al content AlGaN alloys and Ga_2_O_3_ have promise for future RF electronics and power switches. One of the key issues that arises from using these ternary alloys is the low intrinsic thermal conductivity of AlGaN and Ga_2_O_3_ and the low thermal boundary conductance at contacts with these alloys. This requires careful design of the device architecture and layout in order to yield effective heat dissipation pathways for AlGaN systems. In this talk, we present modeling results which demonstrate specific regimes where cooling from the backside or topside of the ultrawide bandgap devices provide the most efficient pathway for heat dissipation. In addition, we present experimental results that demonstrate the effectiveness of the integration of high thermal conductivity dielectrics to enhance the thermal dissipation from these devices. Limitations on the integration of these layers through direct growth or bonding and the thermal resistance of these approaches are discussed as well. Finally, new opportunities to enhance heat transport in these materials using the concept of digital alloy structural ordering are presented.

### 5.2. Anisotropic Thermophysical Properties of Graphite Sheets

Dale Hume * and David Landry

Thermtest, Inc.
* Correspondence: dhume@thermtest.com

**Abstract:** Graphite consists of sheets of graphene held together by van der Waals bonds. The carbon atoms in the graphene sheets are connected by much stronger bonds. This unique structure creates a distinct through-plane direction which is measured to have a different thermal conductivity from the in-plane direction. This measurement is performed on graphite samples of varying thickness using a combined approach involving the transient plane source (TPS) slab method for in-plane properties and transient thermal resistance (TTR) for through-plane thermal conductivity.

### 5.3. Thermal and Mechanical Characterization of Air Bridge Vacuum Field Effect Transistors

Vivek Manipalli ^1,^*, Samuel Graham ^2^ and Damena Agonafer ^1^

1 University of Maryland College Park
2 Georgia Institute of Technology
* Correspondence: vmanepal@umd.edu

**Abstract:** Before the invention of transistors and solid-state semiconductor devices, vacuum tubes were commonly used for signal amplification and current rectification. These were prevalent in the industry for the better part of the early 20th century, with applications in radio, television, radar, and telephones. Solid-state devices, which are smaller, faster, more durable, more efficient, and more economical than vacuum tubes, have replaced them. However, the recent invention of nanoscale vacuum transistors provides an alternative to solid-state transistors. In these devices, electrons flow across a vacuum nanogap from emitter to collector. The electrons can travel at higher speeds in a vacuum than in a solid channel due to fewer collisions, thereby achieving higher current densities. Vacuum transistors are impervious to radiation effects, and can operate at much higher temperatures than traditional solid-state electronics.

### 5.4. Probing New Regimes of Interfacial Heat Transfer with Angstrom-Femtosecond Resolution

Aditya Sood *

Princeton University
* Correspondence: aditya.sood@princeton.edu

**Abstract:** Understanding heat transfer across interfaces is crucial for uncovering the fundamental limits of energy dissipation in nano-electronics. As devices scale down to the atomic level, non-equilibrium effects between electrons and phonons can dominate energy dissipation. Deciphering the physics of thermal transport in such systems requires new metrologies that can directly measure lattice temperature with atomic precision and femtosecond time resolution. I will describe our recent efforts to achieve this via time-resolved diffraction techniques, wherein Debye–Waller effects provide direct measurements of thermal vibrational amplitudes, and consequently lattice temperature. Using this approach on an optically excited van der Waals 2D heterostructure, a new regime of ultrafast energy transfer involving phonon-assisted electron transfer via a layer-hybridized state is discovered [1].

**Reference**

1. Sood et al. Bidirectional phonon emission in two-dimensional heterostructures triggered by ultrafast charge transfer *Nat. Nanotechnol.* **2023**, *18*, 29–35.

### 5.5. Leveraging In Situ Sensors in Laser Additive Manufacturing to Extract Thermal Diffusivity of Thermoelectric Materials Undergoing Rapid Melting and Solidification

Saniya LeBlanc ^1,^*, Bengisu Sisik ^1^, John Middendorf ^2^, Joe Walker ^2^, Kausik Sarkar ^1^

1 The George Washington University
2 Open Additive
* Correspondence: sleblanc@gwu.edu

**Abstract:** Additive manufacturing enables hierarchically structured customized shapes that are unattainable through traditional manufacturing techniques. The advent of additive manufacturing is well studied for structural materials; however, use of this technique for functional materials is nascent, particularly for semiconductor materials with solid-state energy conversion capability. Thermoelectric materials are an example of functional materials that enable conversion of heat into electricity. Studies have shown that thermoelectric devices with non-conventional thermoelectric leg geometries can have improved performance of up to double the power output [1,2], motivating the use of additive manufacturing for thermoelectric devices. Laser powder bed fusion, a laser-based additive manufacturing technique, achieves free-form fabrication of a part by iteratively scanning a laser across thin powder layers that melt and fuse in the chosen scan pattern. Achieving near-full density parts through the rapid and repeated melting of powder material is strongly dependent on the thermal characteristics of the powder, molten, and fully dense material; thus, in situ characterization of thermal properties is needed. This study determines the temperature-dependent thermal diffusivity of bismuth telluride, a well-known thermoelectric material, by using in situ data from a long-wavelength infrared sensor integrated into a commercial laser powder bed fusion tool. Numerical and analytical techniques are used to perform non-invasive thermal characterization during the laser powder bed fusion process. A numerical approach that fits the in situ spatial and temporal temperature data to a solution of the heat diffusion equation was used to determine a thermal diffusivity range for bismuth telluride during the full heating, melting, and solidification process. The extracted thermal diffusivity values are consistent with the values reported in the literature, and they provide a basis for establishing thermal diffusivity for temperatures that have rarely or never been reported before. This approach can inform the process–structure–property relationships for additive manufacturing of multifunctional materials.

**References**

1. Bengisu, S.; LeBlanc, S. The influence of leg shape on thermoelectric performance under constant temperature and heat flux boundary conditions. *Front. Mater.* **2020**, *7*, 595955.
2. Yohann, T.; LeBlanc, S. The impact of thermoelectric leg geometries on thermal resistance and power output. *J. Appl. Phys*. **2019**, *126*, 095101.

### 5.6. The Game of Capacitance and Conductance: How High Thermal Conductivity Phase Change Materials Enable Transient Thermal Mitigation in Pulsed Power Electronics

Adam Wilson *

US Army Research Laboratory
* Correspondence: adam.a.wilson6.civ@army.mil

**Abstract:** Phase change materials (PCMs) have been explored for their use in thermal energy storage and transient thermal mitigation strategies in recent decades, leading to substantial savings in size, weight, power, and cost in the space, industrial, and military sectors. However, conventional phase change materials such as paraffin waxes are limited by their low thermal conductivity, which prevents heat from penetrating far enough into the PCM to make full use of the energy associated with phase change. In this presentation, a series of new high thermal conductivity materials and composites is discussed for their use in transient thermal mitigation applications. Each of these balances thermal conduction with heat capacity and demonstrates the benefits of phase change in controlling transient thermal excursions in high-power systems. Additionally, each has required development of material and/or component thermal analysis strategies, which will be strongly emphasized throughout the topics discussed in the presentation. First, recently completed efforts to develop nickel, titanium, and copper composite structures for thermal energy storage will be discussed. Next, an overview of current work related to development and testing of low-melting-point metals (such as gallium and fields metal) infiltrated into nickel foam matrices will be covered. Finally, the performance impacts of these phase change materials and composites in transient thermal mitigation configurations will be covered, showing (via simulation and experimental testing) that high thermal conductivity phase change materials have a substantial impact on transient temperature rise experienced under high power conditions.

## 6. Session: Nanocalorimetry and Fast Scanning

### 6.1. Effect of Measured Mass versus Estimated Mass on the Specific Thermal Properties of Aluminum as Measured by Nanocalorimetry

Lakshmi Navi Narayan *, Feng Yi, William Osborn and David LaVan

National Institute of Standards and Technology
* Correspondence: lakshmi@uconn.edu

**Abstract:** A major challenge in thermal analysis at the nanoscale lies in determining the sample mass used in the analysis. Sample mass in nanocalorimetry is currently estimated. One method of estimation is from the volume of the material that is deposited as a thin film; however, the density of these films often varies from the bulk value. Another method is to compare the heat capacity or the heat of fusion with conventional calorimetry; however, this property is known to change with heating rates, which are very rapid in nanocalorimetry and considerably slower in conventional calorimetry.

In this work, the mass of a sample was determined using a laser Doppler vibrometry technique, which exploits the vibration of the nanocalorimetry sensor membrane. In this technique, the addition of the sample mass on the sensor membrane causes a shift in the resonant frequency. The resonant frequency is measured via laser Doppler vibrometry and changes in the resonant frequency are correlated with added mass. This sample is then melted. The specific thermal properties from the melting were compared with those from other nanocalorimetry measurements in which the sample mass was estimated.

### 6.2. Preliminary Nanocalorimetry Study of Phase-Change Superlattices

Jie Zhao ^1,^*, Asir Intisar Khan ^2^, Mikhail Y. Efremov ^3^, Zichao Ye ^1^, Xiangjin Wu ^2^, H.-S. Philip Wong ^2^, Eric Pop ^2^ and Leslie Allen ^1^

1 University of Illinois Urbana-Champaign
2 Stanford University
3 University of Wisconsin—Madison
* Correspondence: jiezhao5@illinois.edu

**Abstract:** Phase-change memory (PCM) devices rely on Joule heating of a nanoscale region of chalcogenide material (e.g., Ge2Sb2Te5 or GST) to achieve fast switching between a high-resistance (amorphous) state and low-resistance (crystalline) state. As a result, the substantial switching current density [1] has been a concern for traditional PCM devices based on GST. Recently, a remarkable reduction in power consumption has been reported for PCM devices which incorporate superlattices (SLs), i.e., alternating layers of nanometer-thin chalcogenide films [2,3,4]. However, the switching mechanism of SL-PCM remains unclear, requiring additional thermodynamic insights.

In this work, we use thin-film nanocalorimetry [5,6,7] to probe the phase transition of 65 nm-thick Sb2Te3/Ge2Sb2Te5 superlattices [8]. Nanocalorimetry is a powerful tool for the thermal study of thin-film phase-change materials. It mimics the operation of devices with thin-film compatibility as well as ultrafast scanning rates (110,000 K/s), which mitigates concerns about the transferability of results obtained from model systems to device-level applications. For a 2/1.8 nm/nm Sb2Te3/Ge2Sb2Te5 superlattice, we observed an endothermic transition with ~240 °C depression of temperature and an ~8x reduction in enthalpy compared to melting of GST, providing key thermodynamic insights into the low-power switching of superlattice-based PCMs.

**References**

1. Li, X.B.; Chen, N.K.; Wang, X.P.; Sun, H.B. Phase-Change Superlattice Materials toward Low Power Consumption and High Density Data Storage: Microscopic Picture, Working Principles, and Optimization. *Adv. Funct. Mater.* **2018** *28*, 1803380. https://doi.org/10.1002/adfm.201803380.
2. Khan, A.I.; Daus, A.; Islam, R.; Neilson, K.M.; Lee, H.R.; Wong, H.S.P.; Pop, E. Ultralow–switching current density multilevel phase-change memory on a flexible substrate. *Science* **2021**, *373*, 1243–1247. https://doi.org/10.1126/science.abj1261.
3. Khan, A.I.; Kwon, H.; Chen, M.E.; Asheghi, M.; Wong, H.S.P.; Goodson, K.E.; Pop, E. Electro-thermal confinement enables improved superlattice phase change memory. *IEEE Electron Device Lett.* **2021**, *43*, 204–207. https://doi.org/10.1109/LED.2021.3133906.
4. Khan, A.I.; Wu, X.; Perez, C.; Won, B.; Kim, K.; Ramesh, P.; Pop, E. Unveiling the effect of superlattice interfaces and intermixing on phase change memory performance. *Nano Lett.* **2022,** *22*, 6285–6291. https://doi.org/10.1021/acs.nanolett.2c01869.
5. Lai, S.L.; Guo, J.Y.; Petrova, V.; Ramanath, G.; Allen, L.H. *Phys. Rev. Lett.* **1996**, *77*, 99. https://doi.org/10.1103/PhysRevLett.77.99.
6. Efremov, M.Y.; Olson, E.A.; Zhang, M.; Zhang, Z.; Allen, L.H. Glass transition in ultrathin polymer films: calorimetric study. *Phys. Rev. Lett.* **2003**, *91*, 085703. https://doi.org/10.1103/PhysRevLett.91.085703.
7. Zhao, J.; Hui, J.; Ye, Z.; Lai, T.; Efremov, M.Y.; Wang, H.; Allen, L.H. Exploring “No Man’s Land”—Arrhenius Crystallization of Thin-Film Phase Change Material at 1 000 000 K s− 1 via Nanocalorimetry. *Adv. Mater. Interfaces* **2022**, *9*, 2200429. https://doi.org/10.1002/admi.202200429.
8. Zhao, J.; Khan, A.I.; Efremov, M.Y.; Ye, Z.; Wu, X.; Kim, K.; Lee, Z.; Wong, H.S.P.; Pop, E Allen, L.H. Probing the Melting Transitions in Phase-Change Superlattices via Thin Film Nanocalorimetry. *Nano Lett.* **2023**, *23*, 4587–4594. https://doi.org/10.1021/acs.nanolett.3c01049.

### 6.3. Measurements of Flow-Enhanced Nucleation after Processing of Polyethylene Using Fast-Scanning Calorimetry

Kenneth Kearns *

Dow Chemical Co.
* Correspondence: klkearns@dow.com

**Abstract:** The flow-enhanced nucleation and subsequent crystallization of polyethylene (PE) is a ubiquitous aspect of processing these materials into final articles. Better understanding of the role polymer flow plays on crystallization will allow industry to design better materials for downgauging and recycling. We have developed a new methodology using fast-scanning calorimetry (FSC) whereby the effect of flow on crystallization kinetics in PE can be determined after commercial scale processing. Because of the fast scanning associated with the calorimeter along with the long polymer relaxation timescales, the effect of processing on crystallization behavior can be measured. Initial work utilized a rheometer to process these materials in a controlled way and showed that overall crystallization kinetics increased with shearing rate. We have subsequently observed that linear low-density polyethylene (LLDPE) materials with different molecular architectures respond differently on different blown film lines. These results indicate that polymer flow was different during blown film processing and that the molecular architecture responds to flow in unique ways. Additionally, crystallization data from this FSC methodology allow for the flow-enhanced nucleation parameter associated with non-equilibrium molecular dynamic modeling of crystallization to be determined for the first time.

### 6.4. Spectroscopy Study of PA66 and PA6 under Fast Cooling and High Undercooling Conditions

Xiaoshi Zhang ^1,^*, Alicyn Rhoades ^1^ and John Buzinkai ^2^

1 Penn State Behrend
2 INVISTA Nylon Polymer
* Correspondence: xvz5402@psu.edu

**Abstract:** Polyamide 66 (PA66) and Polyamide 6 (PA6) are crucial engineering plastics for a wide range of applications, and their processing under various conditions, including fast cooling or high undercooling, is of significant industrial interest. In this study, we utilize the flash scanning chip (FSC) technique, which offers precise control over heating and cooling rates up to 4000 K/s, simulating real-life processing conditions. Our previous research has demonstrated successful data collection of spectra, diffractograms and AFM morphology through ex situ experiments after quenching the sample to room temperature post-crystallization. Despite the high cooling rate provided by FSC, which eliminates potential crystallization during cooling, the Brill transition persists in both nylon samples. This presentation will focus on the comparison between ex situ and in situ spectroscopy data collection. By implementing in situ spectroscopy, we are able to achieve a comprehensive polymorphism study that better reflects the true behavior of these materials under various processing conditions.

### 6.5. Structural and Calorimetric Analysis by Combination of Electron Microscopy and Fast Differential Scanning Calorimetry

Jürgen Schawe *

Mettler-Toledo GmbH—Analytical
* Correspondence: juergen.schawe@mt.com

**Abstract:** A device combining scanning electron microscopy (SEM) and fast differential scanning calorimetry (FDSC) has been developed to perform in situ characterization of phase transformations in metastable materials. The potential of this device is demonstrated using a eutectic AuGe alloy as an example. Upon rapid cooling, the alloy forms the metastable crystalline phases β and γ along with an amorphous phase. The changes in microstructure are measured simultaneously with the associated enthalpy changes.

Additional kinetic information on the phase transformations is obtained by ex situ FDSC measurements in a wide scanning-rate range of more than four decades and isothermal measurements in a wide temperature range. We illustrate that the activation energy for the transformation of the metastable γ-phase to the Au α-phase depends critically on the pathway of the phase transition. This effect was further studied in detail by temperature-dependent transmission electron microscopy (TEM) investigations. The thermophysical properties of the glassy phase were determined by FDSC.

### 6.6. Thermodynamics and Kinetics of the Crystallization of Phase Change Materials from Simultaneous Nanocalorimetry and In Situ Microscopy

Isak McGieson ^1^, Feng Yi ^2^, Jim Ciston ^3^, David LaVan ^2,^* and Melissa K. Santala ^1^

1 Oregon State University
2 National Institute of Standards and Technology
3 National Center for Electron Microscopy, Molecular Foundry, Lawrence Berkeley National Laboratory
* Correspondence: david.lavan@nist.gov

**Abstract:** In situ nanocalorimetry synchronized with high framerate transition electron microscopy (TEM) imaging was used to investigate the thermodynamics and kinetics of the crystallization of the phase change materials (PCMs) Ag3In4Sb74Te17 (AIST) and GeTe above their glass transition temperature (Tg). In situ nanocalorimetry provides accurate temperature and energy measurements within the TEM platform, and provides the high heating rates necessary to observe PCM crystallization above Tg. Direct electron detectors capture the crystal growth in the milliseconds before impingement enabling direct measurement of the crystal growth rate. The combined activation energy for nucleation and growth, the activation energy for growth, and enthalpy of crystallization are calculated from the nanocalorimetry measurements. Classical models for nucleation and growth are fit against these data and models that predict the growth rate solely from calorimetry data are compared to the direct growth rate measurements.

## 7. Session: Electronic Packaging Materials

### 7.1. Impact of Conformal Coatings on Thermal Resistance through Thermal Interface Materials

Karl F. Schoch, Jr. *, Nicholas A. Secord, Philip A. Panackal and Curtis D. White

Northrop Grumman Mission Systems
* Correspondence: k.eric.schoch@ngc.com

**Abstract:** Thermal interface materials (TIMs) perform a vital function in electronic assemblies by ensuring efficient heat transfer away from heat-generating components. There are several types of TIMs, including pastes, phase change materials, adhesives, cure-in-place gap fillers, and greases. Frequently, an electronic assembly includes application of a conformal coating at the final step of manufacturing. The conformal coating provides protection during handling of the circuit card and reduces the risk of electrical failure due to formation of conductive dendrites during operation in a humid environment. Designers normally require masking of surfaces prior to coating when there is a TIM applied in a subsequent step. If the masking is unnecessary, this would simplify the manufacturing process.

The purpose of this work was to determine the effect of various conformal coatings on the thermal performance of an assembly having a thermal interface material. The objective herein was to measure the impact of several popular dielectric conformal coatings on the performance of two commonly used thermal interface pads, Henkel Bergquist VO Ultrasoft and Parker-Chomerics Therm-A-Gap G579. Conformal coatings evaluated were a fluoropolymer, two plasma-polymerized coatings, and a polyurethane. Test specimens were fabricated by coating gold-plated copper disks with a conformal coating and then placing a TIM between two coated disks. The thermal diffusivity of the stackup was measured by the flash method at various stages of compression of the TIM. The test results for each material/coating combination were compared with the performance of a stackup without conformal coating.

Test results showed that even though some of these coatings were very thin, the impact on overall thermal resistance may cause a significant increase in temperature through the bondline depending on the heat flux in a given assembly. Moreover, the impact of the coating originates from two factors: (1) the thermal resistance of the coating itself, and (2) the contact resistance at the interface between the TIM and the coating as well as the interface between the coating and the substrate of the coating (i.e., most often the surface of the electronic package or device). Another general observation is that there was considerable variability in the contact resistance from specimen to specimen. Each coating was applied at a typical thickness by the same equipment and process used in production. While both pads are filled silicones, the interaction with the conformal coatings was different, meaning that the impact of a particular coating on heat transfer was not necessarily the same with the two pads.

Approved for Public Release: NG23-1130. © 2023 Northrop Grumman Systems Corporation

### 7.2. Thermal Management and Modelling for Electronics Packaging

Chris Bailey *

Arizona State University
* Correspondence: christopher.j.bailey@asu.edu

**Abstract:** Advanced electronics packaging for system and heterogeneous integration of electronic, electrical, optoelectronic, biological, micromechanical, and sensing components is estimated to grow significantly over the next ten years. These advanced packaging technologies (wafer-level packaging, 3D-IC, embedding, heterogeneous integration) and the recent use of additive manufacturing technologies pose challenges for designers in terms of die–package–system interactions (electrical, thermal, mechanical) that need to predicted at the early stage of package design to ensure the final package meets customer requirements in terms of performance and reliability.

Thermal management and thermo-mechanical modelling are key requirements for electronic packaging engineers. The materials that make up the electronic package which encases semiconductor chips need to be optimized for both thermal performance and thermo-mechanical behaviour to ensure that temperature requirements and resulting thermo-mechanical stresses are met in order to avoid mismatches in the coefficient of thermal expansion.

This paper discusses both active and passive cooling technologies for electronic packaging, with a focus on aerospace applications. Examples include cover modelling for heat sink designs [1] and the physics of failure analysis using physics-informed AI [2] and finite element analysis [3]. We demonstrate how advanced metrology and multi-physics modelling can be used to predict thermal performance and reliability due to thermo-mechanical loading on the package. The presentation details the challenges for design and modelling in terms of electro-thermal co-design (chip–package–system) and multi-physics modelling across length scales.

**References**

1. Santhanakrishnan, M.S.; Tilford, T.; Bailey, C. Multi-objective NSGA-II based shape optimisation of the cross-sectional shape of passively cooled heat sinks. *Int. J. Numer. Methods Heat Fluid Flow* **2022**, *32*, 1025–1045. https://doi.org/10.1108/HFF-10-2020-0656.
2. Stoyanov, S.; Bailey, C. Deep Learning Modelling for Composite Properties of PCB Conductive Layers. In Proceedings of the 23rd International Conference on Thermal, Mechanical and Multi-Physics Simulation and Experiments in Microelectronics and Microsystems (EuroSimE), St Julian, Malta, 25–27 April 2022; pp. 1–7. https://doi.org/10.1109/EuroSimE54907.2022.9758885.
3. Xu, Y.; Xian, J.; Stoyanov, S.; Bailey, C.; Coyle, R.; Gourlay, C.; Dunne, F. A multi-scale approach to microstructure-sensitive thermal fatigue in solder joints. *Int. J. Plast.* **2022**, *155*, 103308. https://doi.org/10.1016/j.ijplas.2022.103308.

### 7.3. Investigation of Electrical Conductivity and Structural Formation of Transient Liquid Phase Sinter Alloys

Gilad Nave * and F. Patrick McCluskey

University of Maryland/College Park
* Correspondence: gnave@umd.edu

**Abstract:** The growing demands of electrification are driving research into new electronic materials. These electronic materials must have high electrical conductivity, withstand harsh environments and high temperatures, and be reliable parts of complete electronic packaging solutions. This study focuses on characterizing the Additive Manufacturing (AM) process of Transient Liquid Phase Sinter (TLPS) alloys in a paste form as candidates for high-temperature and high-power electronic materials. The research focuses on understanding the underlying mechanisms that contribute to the electrical evolution and structural formation of TLPS materials, with a particular emphasis on in situ electrical characterization, electrical percolation, packing densities, and the behavior of the liquid metal phase.

The initial stages of TLPS paste processing, particularly during organic binder burnout and transient liquid phase presence, exhibit interrelated effects requiring standardization and decoupling. Various combinations of paste, organic binders, low-temperature melting particles (LMPs), and heating rates were examined. We developed in situ electrical characterization techniques to monitor the TLPS material’s conductivity evolution during heating up to 300˚C. Subsequently, Environmental Scanning Electron Microscopy (ESEM), Energy Dispersive Spectroscopy (EDS), and structured white light profiling were used to examine the electrical and structural formation of the TLPS traces.

In situ electrical tests revealed rapid evolution of electrical conductivity, with changes of up to seven orders of magnitude within a narrow temperature range. We observed a decrease in material packing density after the LMPs’ melting stage. This phenomenon is attributed to multiple modes of capillary interactions between solid and liquid phase metal particles. Furthermore, diffusion fronts were observed to be contributors to the TLPS material’s rapid structural formation.

The TLPS paste’s initial processing stage and electrical evolution curve are divided into six distinct stages. For steps that show linear relationship with the temperature, the Arrhenius relationship and Linear Mixed Models (LMM) techniques are used to extract associated energies. This analysis provides insights into the underlying mechanisms driving the processing stages.

### 7.4. Impact of Thermal Cycling on Degree of Cure and Physical Properties of Polymers

Kortney Kersten *

Northrop Grumman Mission Systems
* Correspondence: kortney.kersten@ngc.com

**Abstract:** Depending on the end-use temperatures of an assembled system, temperature cycling can greatly affect the physical and mechanical properties of a cured polymer within the assembly. Upon initial curing, polymer properties such as the degree of curing, glass transition temperature (Tg), coefficient of thermal expansion (CTE), and electrical conductivity are measured to determine whether sufficient properties have been achieved and the polymer has been satisfactorily cured. It is well documented that variations in the cure temperature and time can alter these properties; however, little study has been carried out into how these properties change during initial temperature cycling of the parts. Herein, the change in Tg and electrical conductivity is examined for a silver-filled epoxy used to adhere metallic components to printed wiring boards (PWBs). This examination provides a better understanding of how to measure the degree of curing of a composite epoxy material. The investigation highlights the complexity of composite systems in terms of curing and the sensitivity of physical properties with respect to both the curing conditions and short-term aging.

## 8. Session: Rheology

### 8.1. Understand Your Rheometer: Critical Calibration Procedures for Orthogonal Superposition Rheology

Ran Tao * and Aaron Forster

National Institute of Standards and Technology
* Correspondence: ran.tao@nist.gov

**Abstract:** Accurate measurement of the rheological properties of complex fluids is important for scientific research as well as in product development for many industries. Orthogonal superposition rheology, an advanced technique now commercially available for the rheology community, offers a robust method for measuring viscoelastic properties under nonlinear flow. However, given the complicated geometry design, proper end-effect corrections should be applied to the measured viscosities, otherwise significant errors may occur. In this work, we present calibration protocols using Newtonian standards and discuss the results of a shear-thinning fluid. This work provides a technical resource for understanding the geometric constants and identifying the appropriate measurement range (e.g., shear rate, frequency, etc.), including routine rheological methods for proper use of the instrument, which can be beneficial to all rheometer users [1,2].

**References**

1. Tao, R.; Forster, A.M. Calibration Procedures for Orthogonal Superposition Rheology. *J. Vis. Exp*. **2020**, *165*, e61965. https://doi.org/10.3791/61965.
2. Tao, R.; Forster, A.M. End effect correction for orthogonal small strain oscillatory shear in a rotational shear rheometer. *Rheol. Acta* **2020**, *59*, 95–108. https://doi.org/10.1007/s00397-019-01185-5.

### 8.2. Rheological Studies of Polymeric Foam Materials for Mission-Adaptive Law Enforcement Helmet Suspension

Shuang Jin ^1,^*, Linh Pham ^1^, Felipe Santos ^2^, Hossein Bahreinizad ^2^, Sudeesh Subramanian ^2^, Weilong Cong ^2^, Suman Chowdhury ^2^ and Gregory McKenna ^1^

1 North Carolina State University
2 Texas Tech University
* Correspondence: sjin8@ncsu.edu

**Abstract:** Law enforcement officers are susceptible to traumatic brain injuries from both handheld and ballistic projectiles. This requires a new state-of-the-art helmet suspension and padding design to prevent concussion during both linear and angular impacts that occur over a range of rates, from relatively slow to extremely high velocities. Polymeric foam materials have shown promising compressive and shear strength for impact resistance and are widely used in helmet paddings. We have performed dynamic rheology on commercially available military helmet padding foams as well as commercial foam sheets over a range of temperatures, from room temperature to 100 °C, in order to apply time–temperature superposition to study their behavior at low- and high-frequency ranges that correspond to handheld projectiles and ballistic projectiles, respectively. We performed compression and tension measurements of these foams at ambient temperature. Our results show that the current commercial helmet suspension pads do not provide enough protection at high testing rates typical of ballistic impact. A new design to ensure that the suspension system is effective over a wide frequency range is under development.

### 8.3. Beta-Relaxation in Epoxy Thermosets and Its Impact on Polymer Matrix Composite Toughness

Gale Holmes ^1,^*, Mickey Richardson ^2^, Anthony Kotula ^1^ and Jeremiah Woodcock ^1^

1 National Institute of Standards and Technology
2 University of North Texas
* Correspondence: gale.holmes@nist.gov

**Abstract:** Prior to 1975, the preferred matrix for filament wound polymer matrix composites (PMCs) consisted of an epoxy blend composed of diglycidyl ether of bisphenol-A (DGEBA) and bis(2,3-epoxycyclopentyl) ether (Bis-CPE, reactive diluent) cured with a eutectic blend of meta-phenylenediamine (m-PDA) and 4,4′-methylenedianiline (MDA) (e.g., Tonox 60-40 or ZZL 0820). The production of Bis-CPE ceased in 1975, and the diglycidyl ether of 1,4-butanediol (DGEBD, reactive diluent) was determined to be a suitable replacement [1,2]. Although reactive diluents are primarily used to lower the viscosity of the resin during composite processing, it is now known that under the proper cure conditions these flexible additives can be added to a DGEBA/amine network with minimal reduction in the glass transition temperature (Tg) and no loss in modulus, which is important for PMC strength, while increasing the strength and failure strain of the matrix. Recent research by McCarthy suggests that DGEBD has the potential to enhance PMC toughness by suppressing mode 1 matrix crack formation during fiber fracture [3], even though the KIcm and GIcm matrix toughness does not appear to be increased [4]. This suggests that the mode 1 matrix crack suppression which occurs during the dynamic fiber fracture process may be related to the changes in the glassy state molecular dynamics of the matrix resulting from the addition of the reactive diluent. To probe this hypothesis, the molecular dynamics of the β-relaxation process caused by the addition of the reactive diluent are investigated using frequency–temperature sweep (F-TS) rheology measurements.

**References**

1. Chiao, T.T.; Jessop, E.S.; Newey, H.A. An epoxy system for filament winding. *Sampe Q.* **1974,** *6,* 28–32.
2. Chiao, T.T.; Jessop, E.S.; Penn, L.S. Screening of Epoxy Systems for High Performance Filament Winding Applications. Livermore, CA, UCRL-76836, 1975, pp. 1–13.
3. McCarthy, E.D.; Kim, J.H.; Heckert, N.A.; Leigh, S.D.; Gilman, J.W.; Holmes, G.A. The fiber break evolution process in a 2-D epoxy/glass multi-fiber array. *Compos. Sci. Technol.* **2015**, *121*, 73–81.
4. Urbaczewski-Espuche, E.; Galy, J.; Gerard, J.-F.; Pascault, J.-P.; Sautereau, H. Influence of chain flexibility and crosslink density on mechanical properties of epoxy/amine networks. *Polym. Eng. Sci.* **1991**, *31,* 1572–1580.

### 8.4. Natural Building Blocks of Renewable Materials and Rheology for Industrially Relevant Processing

Ngoc Nguyen *

University of Illinois at Urbana-Champaign
* Correspondence: nanguyen@illinois.edu

**Abstract:** The design of novel polymeric materials utilizing natural and biodegradable polymers or renewable materials as alternatives to petroleum-based chemicals is gaining momentum. This approach aims to reduce plastic waste, enhance materials sustainability, and promote plastic circularity. However, the inherent structural characteristics of renewable materials pose challenges in their processing and manufacturing. For instance, cellulose exhibits strong hydrogen bonding systems, rendering it insoluble in regular solvents and non-meltable. Similarly, lignin presents heterogeneous structural units, branching and three-dimensional macromolecules, resulting in diverse melt-processing properties. In this presentation, I will explore potential strategies to manipulate the thermal and rheological characteristics of these natural polymers, thereby improving their processability for a wide range of applications in reinforced thermal plastics, stimuli-responsive materials, and regenerated natural materials using compression molding, additive manufacturing, and solution spinning.

### 8.5. Crystallization of Nifedipine/SiO_2_ Nanoparticle Systems

Bret Tantorno*, Bailey Stroud, Gregory McKenna

North Carolina State University
* Correspondence: bwtantor@ncsu.edu

**Abstract:** Active pharmaceutical ingredients in class II of the Biopharmaceutics Classification System (BCS) show poor solubility, leading to poor bioavailability in the body. One way to resolve this matter is to administer the pharmaceuticals in an amorphous state, which shows a higher solubility than its crystalline counterparts in dilution experiments [1]. The issue is that the amorphous state is a non-equilibrium state that trends toward the metastable supercooled liquid state followed by crystallization. Thus, understanding the kinetics of crystallization can help to mitigate crystallization in amorphous pharmaceutical ingredients (APIs), leading to stable amorphous compounds and improved bioavailability. Here we investigate the impact of SiO_2_ nanoparticles on the stability of the drug nifedipine (NIF), which is a beta blocker used for blood pressure regulation. Our approach to investigating the crystallization kinetics is to study the nucleation and growth of NIF with varying amounts of added SiO_2_ nanoparticles under the hypothesis that the nanoparticles will interfere with the nucleation and/or growth processes by creating a confinement effect that impacts the kinetics and thermodynamics of the material. Furthermore, there is the possibility that the nanoparticles will introduce surface and interfacial effects that interfere with crystallization [2]. Using differential scanning calorimetry, we found two endothermic melting peaks that are at temperatures similar to those of different polymorphic structures of pure NIF. One melting point, at approximately 152 °C, is considered to be a newly discovered polymorph of NIF that has been attributed to nanoconfinement effects [3,4]. To confirm whether the melting point is a new polymorph, X-Ray diffraction experiments were employed to determine the crystalline structure. Furthermore, the crystallization kinetics were followed using dynamic time sweep experiments using rotary rheometry on both pure NIF and the NIF/SiO_2_ system. In the process of constructing a time–temperature transformation (TTT) diagram [5], we were able to show that the onset and completion times for crystallization vary depending on the amount of SiO2, suggesting that the mechanism of crystallization is dependent on particle size, particle concentration, and possibly particle size distribution. We discuss how understanding nanoparticle incorporation into NIF impacts the thermodynamics and kinetics of crystallization and can lead to strategies that improve the long-term stability of APIs.

**References**

1. Dengale, S.J.; Grohganz, H.; Rades, T.; Lübmann, K. Recent advances in co-amorphous drug formulations. *Adv. Drug Deliv. Rev.* **2016*,*** *100*, 116–125. https://doi.org/10.1016/j.addr.2015.12.009.
2. Descamps, M.; Dudognon, E. Crystallization from the amorphous state: nucleation–growth decoupling, polymorphism interplay, and the role of interfaces. *J. Pharm. Sci.* **2014,** *103*, 2615–2628. https://doi.org/10.1002/jps.24016.
3. Yao, C.; Zhang, S.; Wang, L.; Tao, X. Recent advances in polymorph discovery methods of organic crystals. *Cryst. Growth Des.* **2022**, *23*, 637–654. https://doi.org/10.1021/acs.cgd.2c00960.
4. Cheng, S.; McKenna, G.B. Nanoconfinement effects on the glass transition and crystallization behaviors of nifedipine. *Mol. Pharm*. **2019**, *16*, 856–866. https://doi.org/10.1021/acs.molpharmaceut.8b01172.
5. Cheng, S.; McKenna, G.B. Isothermal crystallization and time–temperature transformation of amorphous nifedipine: A case of polymorphism formation and conversion. *Mol. Pharm*. **2021**, *18*, 2786–2802. https://doi.org/10.1021/acs.molpharmaceut.1c00331.

## 9. Session: Advanced Instrumentation and Tandem Techniques

### 9.1. Theory, Practice, and Applications of Microscale Combustion Calorimetry

Richard E. Lyon *

Federal Aviation Administration
* Correspondence: richard.e.lyon@faa.gov

**Abstract:** Approximately 4500 people die from fire each year in the U.S., prompting regulations governing the flammability of plastics used in consumer electronics, public buildings, and transportation. These regulations have created an annual worldwide market of tens of billions of pounds of flame-retardant plastics. In 1999 the Federal Aviation Administration (FAA) developed pyrolysis–combustion flow calorimetry (PCFC) as a tool to screen research quantities (milligrams) of newly synthesized materials for fire resistance in a long-range effort to develop a fireproof aircraft cabin. In 2007, PCFC was commercialized and codified as ASTM D7309: Test Method to Determine Flammability Characteristics of Plastics and Other Solid Materials Using Microscale Combustion Calorimetry by the ASTM D20 Plastics Committee. Hundreds of units have been sold worldwide since that time for flammability screening of plastics, quality control, forensic investigations, product surveillance, and research on the molecular origins of material fire behavior.

The microscale combustion calorimeter (MCC) offers a flexible and convenient laboratory method of combustion analysis in which the atmosphere and heating rate of a milligram sample in the pyrolyzer can be varied independently to control the fuel-to-oxygen ratio, residence time, and temperature of the pyrolysis gases in the premixed plug flow combustor. These operating conditions have recently been optimized for the study of gas phase combustion reactions with FTIR analysis of the toxic combustion products generated over the full range of fire conditions, from over-ventilated (fuel-lean) to under-ventilated (fuel-rich) fires, in order to identify environmentally friendly and flame-inhibiting compounds for plastics that do not increase the toxic potency of the smoke.

A typical MCC test uses a 3∓5 mg sample and a constant heating rate of 1 K/s in an anaerobic environment. The test typically takes 15 min to run and provides flammability parameters that are reproducible (±9%) and repeatable (±3). Several flammability parameters are measured under fire-like (anaerobic pyrolysis) conditions, including the specific heat of combustion of the sample (hc) and its pyrolysis gases (hc,gas), as well as the ignition temperature (Tign) and burning temperature (Tburn) of the solid by isokinetic analysis of the fractional conversion rate history. A molecular combustion model calibrated to bench- and full-scale fire tests shows that the capacity for fire growth (or fire growth capacity/FGC) of a combustible solid initially at ambient temperature T0 is the sum of ignitability and heat release terms, FGC = hc/(Tign − T0) + hc/(Tburn − Tign).

### 9.2. An Advanced Heat Flux DSC Operated in Power Compensation Mode

Jürgen Schawe *

Mettler-Toledo GmbH—Analytical
* Correspondence: juergen.schawe@mt.com

**Abstract:** The instrument presented by Boersma in 1955 can be considered the starting point for the development of disk-type heat flux differential scanning calorimeters (DSC). In this type of DSC, a single furnace contains thermocouples or heat flow sensors with positions for the refence and sample. This type of DSC has been further developed by commercial suppliers and is widely used. Among the advantages of this type of DSC are its high sensitivity and robustness.

The alternative power-compensated DSC technique was first developed by O’Neil in 1964. In this approach, the power required to compensate for heat is measured. The measurement system consists of two small furnaces installed in a cooled metal block. This concept is used for both conventional DSC and fast DSC using chip calorimeters (Flash DSC).

An advantage is the relatively short signal time constant, τ, which is provided not only by the heat transfer conditions in furnace and sample but by the parameter of the controller of the difference temperature.

The new DSC type presented here is based on a conventional heat flux DSC, which has been extended with additional heating elements and temperature sensors for power compensation. This new DSC type was developed to combine the robustness of the heat flux DSC with the signal time constant of a power-compensated DSC. The new developed instrument has electrical power calibration for outstanding accuracy, small time constant for high resolution, improved sensitivity, and excellent baseline stability.

The performance of this device is demonstrated using heat capacity measurements and fast transformations as examples.

### 9.3. Workflow for The Addition of Temperature-Controlled Powder Rheology to Powder Coatings 

**Kimberly Dennis and Keith Coasey**

TA Instruments
* Correspondence: Kimberly_Dennis@waters.com

**Abstract:** Processing of powder coatings involves the complex coupling of granular mechanics, heat transfer, and chemical reactivity, ultimately leading to a deposited film with requisite mechanical and topological characteristics. Optimization of coating properties requires characterization across a broad range of techniques. Thermal analysis allows for characterization of composition, thermal transitions (glass transition, enthalpic recovery, heat of cure), and stability against thermal decomposition. Rheological measurements monitor the physical behavior throughout these transitions, such as the onset of flow, minimum viscosity, and gel point. The new TA Instruments powder rheology accessories with temperature control expand the Discovery Hybrid Rheometer’s capabilities to include measuring the flow behavior of loose powders. The quantitative measurements of powder cohesion, yield strength, and flowability reflect relevant behaviors during storage and processing which may be impacted by variable temperature. The addition of powder rheology measurements to thermal analysis and oscillatory rheology enables a powerful workflow to accelerate formulation development and process optimization.

### 9.4. Pyrolysis-GC/MS as a Method for Quality and Manufacturing Control

Cathy Stewart *

Intertape Polymer Group
* Correspondence: cstewart@itape.com

**Abstract:** Manufacturers often depend on purchased adhesives, polymers, resins, films, and organic additives to manufacture their products. What is the best method for understanding variations in the quality of purchased and finished products? While common analytical tools such as DMA, DSC, TGA, and FT-IR can provide general information about raw materials or finished products, they are often unable to detect subtle batch-to-batch chemical variations. By using pyrolysis coupled with GC/MS, even minor differences may become apparent. Pyrolysis-GC/MS is an important tool for use in the quality control of incoming raw materials, the evaluation of raw material substitutions, and in troubleshooting finished products.

### 9.5. Battery Cycler Microcalorimeter Solution: Unlocking a New Dimension in Battery Research

Calliste Scholl * and Jeremy May

TA Instruments
* Correspondence: calliste_scholl@waters.com

**Abstract:** Screening new battery cell chemistries using traditional electrochemical methods is a time-prohibitive process that significantly slows the pace of research. These methods involve cycling the cell until signs of degradation or sufficient capacity fade are evident, typically taking months to complete. In operando isothermal microcalorimetry is an established technique for measuring the activity of parasitic (or non-reversible) reactions during charge cycling [1,2,3,4]. Parasitic reactions are a blanket term for any side reactions that occur within a battery, including solvent breakdown, lithium plating, self-discharge reactions, and growth or decomposition of the solid electrolyte interphase. Battery formulations with high parasitic heat are strongly correlated with early cell failure. Combining electrochemical methods and microcalorimetry allows for rapid and effective battery cell screening accomplished with the Battery Cycler Microcalorimeter Solution. 

Compared to electrochemical cycling, which often takes months, the average parasitic heat over the full voltage range can be determined in 1–2 weeks. If a narrow voltage range is of interest, the experiment time can be reduced to 2–3 days. This method involves slow galvanostatic cycling at isothermal conditions with simultaneous measurement of heat flow, voltage, and current. The parasitic heat is isolated from the total heat flow using the Integration–Subtraction method, with calculations performed automatically by the software [1]. The Coulombic efficiency is measured per cycle, typically showing an inverse relationship to the parasitic heat. As a screening tool, these data can be used to select the battery formulations most likely to meet performance goals in long-term cycling experiments, rather than wasting valuable time and lab space on a trial-and-error approach. In operando electrochemical microcalorimetry is a powerful tool for investigating the efficiency of new cell chemistries, electrolyte formulations, and studying degradation rates in a fraction of the time compared to traditional methods. The solution described here integrates these two methods together to offer the user a turn-key solution, thereby reducing downtime and maximizing productivity.

**References**

1. Krause, L.J.; Jensen, L.D.; Chevrier, V.L. Measurement of Li-ion battery electrolyte stability by electrochemical calorimetr. *J. Electrochem. Soc*. **2017**, *164*, A889–A896.
2. Krause, L.J.; Jensen, L.D.; Dahn, J.R. Measurement of parasitic reactions in Li ion cells by electrochemical calorimetry. *J. Electrochem. Soc.* **2012**, *159* A937–A943.
3. Burns, J.C.; Kassam, A.; Sinha, N.N.; Downie, L.E.; Solnickova, L.; Way, B.M.; Dahn, J.R. Predicting and extending the lifetime of Li-ion batteries. *J. Electrochem. Soc*. **2013**, *160*, A1451.
4. Logan, E.R.; Louli, A.J.; Genovese, M.; Trussler, S.; Dahn. J.R. Investigating parasitic reactions in anode-free Li metal cells with isothermal microcalorimetry. *J. Electrochem. Soc*. **2021**, *168*, 060527.

## 10. Session: Kinetics

### 10.1. The Kinetics of the Electron Beam Synthesis of Polyvinylpyrrolidone and Polyethylene Glycol Hydrogels

Mohamad Al-Sheikhly ^1,^*, Zois Tsinas ^2^ and Aiysha Ashfaq ^1^

1 University of Maryland College Park
2 Theiss Research
* Correspondence: mohamad@umd.edu

**Abstract:** Hydrogels (nano -gels and macro-gels) that contain alcohol, amino, and carboxyl groups have been successfully used in art restoration. One of the major obstacles of the traditional synthesis of these nanogels and macro-gels is the presence of the remaining residues from the synthesis such as solvents, crosslinking agents, and catalysts. Ionizing radiation, such as that from electron beams, has been successfully used in the synthesis of macro- and nanogels for various applications, and offers a great advantage in that it does not require additional organic solvents, crosslinking agents, or catalysts, only the addition of energy to the systems in terms of high-energy electrons from electron beam accelerators.

We are investigating the radiation-induced synthesis of functionalized polymer nano-hydrogels that can serve in art restoration. The latest results on the synthesis and kinetic analysis of poly(vinyl pyrrolidone) (PVP) nanogels in dilute aqueous solutions using electron beams, particularly at high temperatures, is presented herein. At temperatures above 60 °C, PVP chains start to collapse, decreasing the average hydrodynamic radius (Rh) from 23 at 20 °C to 15.6 nm at 80 °C due to the disruption of polymer–water hydrogen bonds. The collapsed form of the PVP molecules enhances the intra-crosslinking reactions of the radiolytically produced free radicals, leading to a further decrease in average Rh to a value of 14 nm γ-ray irradiation with 10 kGy. The nano-gel structure was synthesized using pulsed electron beam irradiation at high repetition rates, giving rise to a high intra-chain yield of multiple free radicals. These free radicals enhance the intra-crosslinking reactions, leading to the formation of smaller nanogel molecules with an average Rh value of 12 nm at 300 pulses per second. At high pulse repetition rates, the intramolecular crosslinking reactions of the carbon-centered free radicals are preferred; this effect is enhanced at higher temperatures. While high dose rate pulses enhance the intramolecular crosslinking, low dose rate pulses and the extended shape of the PVP molecules favor intermolecular crosslinking, leading to the synthesis of hydrogels with various sizes. In general, in uniform distributed systems such as monomer solutions the distribution of the reactant second-order kinetics is characterized by simple linear dependence of 1/C (or the corresponding absorbance, 1/A) on time. However, dilute polymer solutions follow second-order decay kinetics, which can be characterized in terms of a time constant t = 1/2 kC, corresponding to a biomolecular reaction of a radical reacting with another radical. A typical example of this case is the pulse radiolysis experiment of N_2_O-saturated aqueous solutions of PVP. 

### 10.2. Decay Kinetics of Long-Lived Polyenyl Radicals in Highly Crystalline Ultra-High Molar Mass Polyethylene (UHMMPE) Fibers

Amanda Forster ^1,^*, Zois Tsinas ^2^ and Mohamad Al-Sheikhly ^3^

1 National Institute of Standards and Technology
2 Theiss Research
3 University of Maryland College Park
* Correspondence: amanda.forster@nist.gov

**Abstract:** To improve properties such as thermal conductivity, low temperature thermal strain, and creep resistance of ultra-high molar mass polyethylene (UHMMPE) fibers, researchers have previously undertaken efforts to crosslink these fibers using irradiation. Ionizing radiation is commonly used to crosslink bulk UHMMPE in other applications, such as artificial joints. However, UHMMPE fibers differ from bulk UHMMPE in that they have a higher crystallinity (approximately 85% to 90%) and are highly oriented during manufacturing, meaning that the amorphous fraction of the UHMMPE fibers is highly ordered as well. Several experiments were conducted to crosslink UHMMPE fibers using both low dose rate (gamma) and high dose rate (electron beam) irradiation, all in the absence of oxygen. In all cases, the tensile strength of the fiber was greatly reduced by the irradiation. The oxidation index was measured for the irradiated samples, and oxidation was not found to play a major role in the reduction of tensile strength in the fibers after irradiation. While this work did not achieve the desired result of improving the mechanical properties of the UHMMPE fibers, a significant result was found. The electron paramagnetic resonance (EPR) spectrum of the UHMMPE fibers was measured shortly after irradiation, and a mixture of allyl and alkyl radicals were detected. The irradiated samples were stored in dark ambient conditions for at least six years, then reexamined using EPR for free radical characterization. Surprisingly, the gamma-irradiated samples showed clear evidence of long-lived polyenyl radicals present in the material. Free radicals are very reactive species that typically migrate to the surface of the crystalline domain and decay in a relatively short time through various reactions in the amorphous regions. It is hypothesized herein that due to the high crystallinity and large anisotropy of the highly drawn UHMMPE fiber, the polyenyl radicals were trapped in the crystal phase and were unable to migrate and decay. To test this hypothesis, samples of the irradiated fibers were first heated to temperatures above the alpha relaxation and then above the melting point of polyethylene, and EPR measurements were taken to observe the decay kinetics of the free radicals. The results showed that while the polyenyl radical signal persisted below the Tm, it was rapidly eliminated upon melting of the crystals. The observed free radical decay constant (attributed to second-order radical-radical bimolecular recombination) was an order of magnitude higher at 165 °C than at 80 °C. These experiments support the hypothesis that the long-lived polyenyl radicals are trapped in the crystalline region of the polyethylene fibers.

### 10.3. Kinetic Modeling of Competitive Epoxy Curing Reactions

Steve Sauerbrunn *

University of Delaware—CCM
* Correspondence: sauerbru@udel.edu

**Abstract:** Epoxy curing is often modeled as a single-step reaction. Sometimes, however, the curing chemistry is a sequential two-step mechanism. The work reported here involves the curing of Solvay 977-3 film. The best kinetic model is a two-step mechanism in which the two reactions are in competition for the unreacted epoxy. This kinetic model can be used to predict the extent of curing for any time–temperature profile. A good kinetic model allows simulation of the final curing to address any changes to the manufacturer’s prescribed curing profile.

### 10.4. Preliminary Development in the Synthesis of Alumina-Acrylic Polymer Nanoparticles for Immobilizing Chloride Ion Transport in Concrete

Aiysha Ashfaq *

University of Maryland College Park
* Correspondence: aiysha.ashfaq28@gmail.com

**Abstract:** Restricting the diffusion of chloride ions from reaching the steel reinforcements in concrete structures is an effective method of preventing corrosion Therefore, there is significant research into ways to increase the chloride binding capacity of concrete. Nano-alumina additives are of increasing interest, as they have previously been shown to successfully chemically bind free chloride ions. Even so, optimal concentrations of nanoalumina are not well established. This is due to the propensity of nanoparticles to agglomerate into clusters at high concentrations, which lowers the specific area available for binding. This research seeks to avoid this problem by encapsulating the particle within a semipermeable polymeric nanogel layer.

The encapsulation process involved radiolysis of polyacrylic acid (PAA) in an aluminum chloride aqueous solution by a high energy (11 MeV) electron beam. Dynamic Light Scattering (DLS) measurements establish that the hydrodynamic radius of the Al-PAA nanoparticles is tunable by varying irradiation conditions (dose rate, dose, energy, solution chemistry, and temperature). The nanogels had a significantly smaller hydrodynamic radius (30–40 nm) than bare alumina nanoparticle clusters (200 nm), indicating improved dispersion. This is confirmed by Zeta potential measurements. The nanoparticles were characterized by Fourier Transform IR Spectroscopy (FTIR}, X-Ray Diffraction (XRD), and Transmission Electron Microscopy (TEM).

### 10.5. Epoxy–Amine Curing Kinetics in a Hierarchical Carbon Nanotube Grafted Fiber-Reinforced Polymer Composite

**Aaron Forster, Ajay Krishnamurthy, Ran Tao**

National Institute of Standards and Technology
* Correspondence: aaron.forster@nist.gov

**Abstract:** Carbon nanotube (CNT)-grafted fiber-reinforced polymer composites (FRPs) showcase hierarchical nano- to macro-scale structural reinforcements that result in improved mechanical and electrical properties over traditional FRPs. Direct attachment of CNTs to fiber surfaces allows control of the volume fraction of interfacial CNTs and avoids complications that arise in the resin transfer processes used for FRP manufacture. Often, small macromolecules are used to optimize CNT deposition or improve interfacial adhesion between the macroscale fibers, CNTs, and matrix resins. For our studies, a hyperbranched polyethyleneimine (PEI) was used to both aid CNT deposition and as an active participant in the epoxy curing reaction. Considering the ability of nanofillers to modify both polymer relaxation and curing reactions, it is important to understand how PEI and CNTs modify interfacial properties beyond electrical conductivity or mechanical reinforcement. Previously, we used both bulk and nanoscale characterization techniques, such as dynamic mechanical thermal analysis (DMTA) and elastic neutron scattering experiments, to establish that the heterogeneous fiber–CNT–polymer interphase broadens the viscoelastic relaxation characteristics and polymer molecular dynamics without decreasing the glass transition temperature. Here, we decouple the role of amine functionality through controlled studies of the epoxy–amine curing process. Dynamic and isothermal experiments are conducted using differential scanning calorimetry to develop an understanding of the curing process. We find that hyperbranched PEI affects the overall interface cure kinetics even when added at small mass fractions such as 0.4%. 

### 10.6. The Role of Impurities on Epoxy–Amine Reaction Kinetics

**Gale Holmes, Anthony Kotula, Jeremiah Woodcock, Stephan Stranick**

National Institute of Standards and Technology
* Correspondence: gale.holmes@nist.gov

**Abstract:** Between 1946 and 1959, various works have investigated epoxy–amine (E-A) reaction kinetics in dilute aqueous or dilute alcoholic solutions. From this research, E-A reaction kinetics have been determined to be second-order. It is now well established from the 1961 research of I.T. Smith [1] that the E-A reaction kinetics are autocatalytic and proceed through a termolecular hydrogen-bonded transition state. Thus, impurities that contain hydroxyl groups (i.e., alcohols) or the presence of adventitious hydroxyl groups (e.g., water) accelerate the E-A reaction. Because the initial E-A autocatalytic reaction rate is accelerated with increasing concentration of hydrogen-bond donor molecules, the earlier aqueous and alcoholic research results are now known to be pseudo-second order. This fundamental knowledge has formed the basis for understanding the reaction of bis-epoxides (e.g., diglycidyl ether of bisphenol-A (DGEBA)) with diamines such as meta-phenylenediamine (m-PDA) to make thermoset resins [2]. Noting that thermoset resins are widely used in the manufacture of fiber reinforced composites, reactive diluents are often added as a processing aid to reduce the resin’s initial viscosity and facilitate composite manufacture. In this presentation, we show that the hydroxyl-containing DGEBA impurities accelerate the DGEBA/m-PDA reaction kinetics, as expected from Smith’s autocatalytic reaction scheme. However, when the reactive diluent diglycidyl ether of 1,4-butandiol (DGEBD, 60 mass % purity), which contains numerous hydroxyl-containing oligomers, is added to the DGEBA/m-PDA reaction mixture, the reaction kinetics are not accelerated, instead being dramatically reduced. By integrating a novel mechanophore/molecular probe [3] into the E-A network and using rheo-Raman spectroscopy to investigate the curing behavior [4], the retardation of the reaction kinetics was traced to the hydroxyl-containing DGEBD impurities.

**References**

1. Smith, I.T. The mechanism of the crosslinking of epoxide resins by amines. *Polymer* **1961***, 2*, 95–108.
2. Dusek, K.; Ilavsky, M.; Lunak, S. Curing of epoxy resins. I. Statistics of curing of diepoxides with diamines. *J. Polym. Sci. Part C-Polym. Symp.* **1975**, *53*, 29–44.
3. Woodcock, J.W.; Stranick, S.J.; Kotula, A.P.; Chen, S.H.; Engmann, S.; Gilman, J.W.; Holmes, G.A. Reaction-Induced structural and compositional heterogeneity in amine-cured epoxy/epoxy thermosets: Visualization of heterogeneity using fluorescence lifetime imaging microscopy (FLIM). *Polymer* **2023**, *273*, 125826.
4. Kotula, A.P.; Woodcock, J.W.; Gilman, J.W.; Holmes, G.A. A cure kinetics investigation of amine-cured epoxy by Rheo-Raman spectroscopy. *Polymer* **2023**, *278,* 125967.

### 10.7. TuFF Composite Production after Determining Curing Conditions of Hexcel M77 Fast-Curing Resin through Thermal Analysis

Tekin Ozdemir *

University Of Delaware Center For Composite Materials
* Correspondence: tozdemir@udel.edu

**Abstract:** The high-performance University of Delaware patented material TuFF (Tailored Universal Feedstock for Forming) achieves properties equivalent to the best continuous fiber composites used in many applications, including aerospace. TuFF is the world’s strongest short-fiber composite material that can be converted into complex shapes. Another characteristic that is necessary to gain acceptance for automotive applications is the ability to process the material at similarly fast production rates to metals.

In order to demonstrate the ability to rapidly form or cure the TuFF material, Composites Automation LLC (CA) has procured resin film forms of snap cure resin systems. One of the systems that CA is currently investigating for utilization with the TuFF material is Hexcel’s M77 epoxy. M77 resin cures in 90 s at a temperature of 150 °C. The way that CA is utilizing M77 is to first prepreg the TuFF layup with M77 resin, followed by rapid curing. To develop the process conditions for both prepreging the TuFF material with the M77 resin and determining the conditions required to rapidly cure the prepreg, the curing kinetics of the M77 resin film were determined. These data were obtained through a series of non-isothermal DSC heating ramps carried out at different heating rates in the Netzsch DSC. These rates included 2, 5, and 10 °C/min. The data were imported into Netzsch Neo kinetics software, where model-free Friedman analysis was used to determine the average energy of activation-(Ea) and average pre-exponential factor (-A). These values were used as the seed values for fitting a model to the DSC data.

Prior to molding, consolidation, and curing of TuFF-based prepreg into a part, the epoxy resin must be infiltrated into the dry TuFF preform. Typically, this is done by laying up a sequence of the dry preform and resin film in the correct proportions needed to achieve a desired fiber volume fraction, then applying heat and pressure to the layup in order to allow the resin to completely wet out the dry fiber. Ideally, the resin must be heated to the point where it will flow freely but not so high that significant curing occurs. To determine the ideal temperature range for resin impregnation of the TuFF preform, a non-isothermal Parallel Plate Rheology experiment was carried out. A final DSC confirmation run showed that with a ~3% difference in the overall heat of polymerization, minimal progression of the cure state for recommended temperature range is achieved.

As a result, it is shown that an automotive-grade composite can be produced in a very short time using fast-curing M77 resin and TuFF material in the same matrix.

### 10.8. A Single-Molecule Approach to Thermokinetic Analysis Using DNA Nanotechnology

Joseph Robertson ^1,^*, Tadas Penkauskas ^1^, Shankar Haridas ^1^, Addhyaya Sharma ^1^ and Joseph Reiner ^2^

1 National Institute of Standards and Technology
2 Virginia Commonwealth University
* Correspondence: joseph.robertson@nist.gov

**Abstract:** Nanopore sensors are emerging as a critical tool to study biopolymers and biopolymer interactions. A single nanopore is embedded in a dielectric membrane separating two electrolyte reservoirs, and the ionic current is measured when an electric field is applied across the membrane. The ionic current is interrupted when individual molecules partition into the nanopore, creating a resistive pulse which can be decoded to produce a measure of the size, shape, structure, dynamics, and chemical interactions of the molecule [1]. Room-temperature measurements produce rich datasets that hint at the molecular details of the interactions that drive nanopore sensing. However, detangling the complex chemical interactions involved in nanopore sensing requires measurements over a wide range of temperatures [2]. We explore temperature control over several temporal and length scales, including uniform heating of the bath [3], diffraction-limited laser control, and plasmonic control [4,5]. We are developing hybrid structures with DNA nanotechnology that allows control of localized surface plasmon resonance objects, such as gold nanoparticles, in order to generate precise and controllable thermal gradients at and across the nanopore. By incorporating devices that employ rapid optical temperature control, we envision the ability to both probe and modify the free energy environment for analyte in the pore. This provides two important results: the ability to better understand molecular interactions with and within the nanopore, and the ability to use this improved understanding to identify these molecules for sensing applications. We will report on recent progress of plasmonic modification protocols and preliminary results from these modified nanopore devices.

**References**

1. Robertson, J.W.F.; Ghimire, M.L.; Reiner, J.E. Nanopore sensing: A physical-chemical approach. *Biochim. Biophys. Acta BBA—Biomembr*. **2021**, *1863*, 183644. https://doi.org/10.1016/j.bbamem.2021.183644.
2. Angevine, C.; Robertson, J.W.; Dass, A.; Reiner, J.E. Laser-based temperature control to study the roles of entropy and enthalpy in polymer-nanopore interactions. *Sci. Adv.* **2021**, *7*, eabf5462. https://doi.org/10.1126/sciadv.abf5462.
3. Jung, Y.; Bayley, H.; Movileanu, L. Temperature-responsive protein pores. *J. Am. Chem. Soc.* **2006**, *128*, 15332–15340. https://doi.org/10.1021/ja065827t.
4. Seol, Y.; Carpenter, A.E.; Perkins, T.T. Gold nanoparticles: enhanced optical trapping and sensitivity coupled with significant heating. *Opt. Lett.* **2006**, *31*, 2429–2431. https://doi.org/10.1364/OL.31.002429.
5. Reiner, J.E.; Robertson, J.W.F.; Burden, D.L.; Burden, L.K.; Balijepalli, A.; Kasianowicz, J.J. Temperature Sculpting in Yoctoliter Volumes. *J. Am. Chem. Soc*. **2013**, *135*, 3087–3094. https://doi.org/10.1021/ja309892e.

### 10.9. A Physical Basis for Kinetic Compensation

Richard E. Lyon *

Federal Aviation Administration
* Correspondence: richard.e.lyon@faa.gov

**Abstract:** The rate of a single-step chemical reaction da/dt is proportional to the product of a rate constant k(T) = Aexp[-E/RT] and a reaction model f(a) in the Arrhenius form of the reaction rate law. The Arrhenius kinetic parameters Ej and Aj for N similar reactants, conditions, models, methods of analysis, or measurement errors are highly, positively, and linearly correlated as ln[Aj] = ln[k0] + Ej/RT0 if an equal (iso) kinetic temperature T0 for the reaction exists. This effect is called kinetic compensation (KCE), because a decrease in E is offset (compensated) by an increase in A such that k(T) is unchanged. The compensation effect has been the subject of more than 50,000 publications over the past 100 years, with no consensus opinion reached about the physical significance of the correlating parameters T0 and k0. In this paper, we postulate that although the total system is not in equilibrium during the chemical reaction(s), there exists within a small mass element a state of local equilibrium such that the local entropy is the same function of the macroscopic properties as for real equilibrium. In this case, there exists a local equilibrium temperature T0 = ∆H°/∆S° and a constant k0 that is the reciprocal of a characteristic time and reconciles the kinetic compensation effect (KCE) with the isokinetic relationship (IKR), i.e., the intersection of Arrhenius lines at 1/T0 in a plot of ln[k] versus 1/T. The proposed physical basis for kinetic compensation is supported by qualitative agreement between ∆H° and ∆S° as calculated from standard enthalpies and entropies of formation and the ensemble averages of Ej and Aj for thermal decomposition of organic peroxides, calcium carbonate, and poly(methyl-methacrylate).

### 10.10. Kinetic Predictions for Multi-Step Chemical Reactions in Large Volumes

Elena Moukhina *

NETZSCH Geraetebau GmbH
* Correspondence: elena.moukhina@netzsch.com

**Abstract:** The small samples in DSC measurements have no significant temperature gradient, making them suitable for kinetic analysis. Simple kinetics software can simulate the rate of chemical reactions for two limiting cases. The first case has two conditions: (1) no temperature gradient because of infinite heat transfer in the sample; and (2) a controlled sample temperature due to infinite heat loss to surrounding. The second limiting case is pure adiabatic heating without any heat loss to surrounding media.

However, in the chemical industry, as well as in the storage and transport of highly energetic materials, the heat transport and heat loss are between these two cases, and for safe conditions it is necessary to carry out simulation of non-constant temperatures in a reacting volume. The areas with higher temperature experience faster reaction and more intensive heat production in the case of exothermal reaction. The increase in the temperature leads to the further heating of these local areas, and can serve as hot spots for thermal explosion. For reactions with lower thermal effects the areas with higher temperature have a higher reaction rate and degree of conversion. This is the reason for the different physical properties of materials at different coordinate points, which appears as shrinkage during sintering or curing, producing mechanical stresses and having a negative influence on product quality.

While many software solutions can calculate the heat transfer, they have problems with complex multi-step chemical reactions where thermal effects are present. Usually, such systems work for model-free kinetics with a single kinetic equation or for models with one or two steps in which all kinetic parameters are known.

Today, new Thermal Simulation software contains both input data in the form of chemical parameters (e.g., the kinetic model from Kinetics Neo software) and the physical parameters such as the temperature-dependent heat capacity, density, and thermal conductivity of the reacting material. It is completely compatible with NETZSCH Kinetics Neo Software and with both model-free and model-based approaches. For model-based approaches there are no limitations on the number of individual reaction steps or connections between them, including independent, competing, or consecutive ones. The additional input parameters include the container materials which can be different for each plane of the reactor geometry, as well as different surrounding media, such as air on the top, water on the side, and ground on the bottom. The surrounding temperatures can be different for different geometry planes.

The simulation results consist of the time-dependent and coordinate-dependent properties such as the temperature, concentrations of all reactants, conversion, and reaction rate. A live presentation of the software using the example of a simulation of an exothermal material will be provided.

### 10.11. A Novel Method for Improving the Reliability of Simulations of the Behavior of Energetic Materials Based on Kinetic Analysis

Bertrand Roduit *

AKTS SA
* Correspondence: b.roduit@akts.com

**Abstract:** The kinetic parameters of the decomposition of materials are generally computed by applying advanced kinetic analysis of conventional non-isothermal DSC experiments. However, the more insightful considerations of this standard procedure may lead to doubts about whether the kinetic parameters of the decomposition obtained from the data collected at high temperatures are representative enough for the low-temperature range at which the shelf life needs to be determined. One cannot exclude the scenario that extrapolation of the thermal behavior of the materials from the high- to low- temperature range may not be precise enough. The reliability of kinetic analysis increases when the temperature of the experimental domain is not much distant from the storage temperature of investigated materials. However, this evident approach is very time-consuming and challenging from a practical point of view. At lower temperatures and/or low heating rates, the intensity of the DSC heat flow signals is very often below the sensitivity of commercial thermoanalyzers. The thermal behavior at low storage temperatures can be monitored only by sensitive, however expensive, isothermal calorimeters such as TAM.

A novel kinetic workflow is proposed in the presented study to overcome this general problem, which has to be considered during the kinetic analyses of all compounds. Its main feature is merging non-isothermal heat flow data obtained in conventional DSC systems at higher temperatures with sparse isothermal data collected at lower temperatures.

The preliminary kinetic analysis uses the non-isothermal data measured at different heating rates, e.g., with 0.25 K/min < b < 8 K/min. Such data collection in the high-temperature domain requires a relatively short collection time. Obtained kinetic parameters are used to simulate the reaction course for isothermal experiments in the moderate-temperature range (for a duration of 0 < t < e.g., 60 days). Such simulation allows for designing the temperature range of low-temperature isothermal experiments. During these experiments, the decomposition of the energetic materials will occur with a heat flow too small to be measured by conventional DSC. However, conventional DSC runs allow quantifying the decomposition extents. The determination of decreasing reaction heat (proportional to the integral intensity of the DSC peak) may be used to quantify the decomposition degree, which, in turn, will allow the kinetic analysis based on sparse isothermal data. 

The proposed procedure results and experimental data elaboration will be discussed during the presentation. As an example, the small calibre type of propellant was used. The material was investigated non-isothermally (0.25–8 Kmin^−1^) by differential scanning calorimetry (DSC) in the range of RT-270 °C at different heating rates and isothermally over 60 days at 105, 110 and 115 °C.

### 10.12. Decomposition of Durite SC-1008 Phenolic Resin Through Thermogravimetric Analysis and Differential Scanning Calorimetry

Perla Salinas ^1,2,^*

1 Sandia National Laboratory
2 Theiss Research
* Correspondence: pasalin@sandia.gov

**Abstract:** Phenolic resin has been widely used and researched for Thermal Protection Systems (TPS) due to its inherent properties such as high strength, high toughness, good thermal stability, electrical isolation, and adhesion properties. However, after exposure to elevated temperatures these designed properties begin to change as the microstructure is altered via additional crosslinking and degradation. It is well known that the processing conditions of the resin can have an effect of the thermal stability of the cured phenolic material. This study demonstrates the measured thermal decomposition of Durite SC-1008 phenolic after being heat treated under two different procedures. The decomposition of the phenolic resin is monitored using Thermogravimetric Analysis (TGA) and Differential Scanning Calorimetry (DSC). These results show that the varying postprocessing effects the stability of the phenolic polymer and can be tuned to extend the service life of the material.

### 10.13. Self-Actuating Mori–Thai Composite Silk Bilayer Materials

Kyle Printon and Xiao Hu *

Rowan University
* Correspondence: hu@rowan.edu

**Abstract:** This study investigates the complex interactions between silk fibroin proteins produced from two different species of silkworms, aiming to generate a controllable bilayer actuation. The passive layer is composed of Bombyx mori (Mori) white silk fibroin, while the active layer is a composite material made of varying ratios of Mori and Thailand (Thai) yellow silk fibroins. Macroscale images of the cross section and surface of each sample ratio were taken via scanning electron microscopy (SEM). The protein spectra of the composites were generated through Fourier transform infrared spectroscopy (FTIR) to determine protein composition as well as the hydrophobicity of each sample. Contact angle was conducted to help determine the hydrophobicity of each sample as well as the swelling properties to design a material with controllable contraction due to swelling and drying. Differential scanning calorimetry (DSC) and thermal gravimetric analysis (TGA) were both conducted to gain further insight on the thermal properties of the material such as degradation and transition temperatures. Additionally, the contraction-based actuation of different bilayer films was tested in an oven to determine the maximum bend angle and the time taken to achieve it. The final design is a bilayer film with the optimal ratios of each silkworm species to enhance actuation angle and speed while maintaining a controllable deployment.

### 10.14. Synthesis, Purification, and Characterization of N,N’-Di(1-naphthyl)-N,N’-diphenylbenzidine (NPB): Part II

Andrew McGhie *, Gilbert J. Sloan and Steven J. Szewczyk

University of Pennsylvania
* Correspondence: mcghie@lrsm.upenn.edu

**Abstract:** This presentation is a follow-up on the poster of the same name that we presented at the 48th NATAS Conference in Cleveland, OH in 2022. Further purification and characterization have been carried out on NPB, a proton transport medium used in OLED devices. Purity has been improved by extensive zone refining and characterization by DSC, FTIR, and LC-MS. Although this material readily forms glass on rapid cooling, significant zone refinement has been found to take place at the glass–liquid interface, which was unexpected. This paper is presented in tribute to our colleague and lead author, Gilbert J. Sloan, who initiated this project, and who died on 24 May 2023 at 94.

## 11. Session: 3D-Printing/Additive Manufacturing

### 11.1. Inter-Filament Fusion Defects in Embedded 3D Printing

Leanne Friedrich *

National Institute of Standards and Technology
* Correspondence: leanne.friedrich@nist.gov

**Abstract:** Extrusion-based 3D printing techniques are among the most versatile ways to fabricate complex structures. These techniques span a materials space ranging from soft hydrogels, colloids, and polymers to ceramic slurries, carbon fiber reinforced epoxies, and liquid metals. They encompass a large design space as well, allowing for customization of material properties and designed anisotropy along the toolpath. Direct ink writing (DIW) is the most common extrusion-based 3D printing technique. In DIW, the nozzle writes continuous filaments onto a substrate and the structure is then solidified after deposition. DIW requires inks with a yield stress such that the ink behaves as a low-viscosity liquid while it is passing through the nozzle and as a solid between deposition and curing. However, many applications, such as bioprinting and printing of soft polymers, require low-viscosity inks. Embedded 3D printing (EMB3D) makes these materials printable. In EMB3D, a nozzle is embedded into a support bath and writes continuous filaments. Because the bath is a yield stress fluid, it yields locally around the nozzle, allowing the nozzle to traverse and extrude material, and then solidifies when the nozzle is gone, holding the printed structure in place. In addition to expanding the printable space into low-viscosity materials, EMB3D enables greater flexibility than DIW in toolpaths and part design, as the nozzle is no longer restricted to layer-by-layer printing and can instead move in any direction.

The defects that emerge in EMB3D are unique from those in DIW, and require separate consideration. For example, while DIW filaments tend to be short and wide, in EMB3D single filaments extruded out of cylindrical nozzles often exhibit non-circular cross-sections, with a sharp top edge and a tall and narrow aspect ratio [1]. Similarly, while interfilament contact tends to be simply achieved in DIW, interfilament contact in EMB3D is non-trivial. Because the nozzle must displace support material rather than air, EMB3D is vulnerable to poor fusion, as support material becomes trapped between filaments. When writing a second line next to an existing line, the nozzle can introduce new defects to the original line, including displacement, leading to inaccurate positioning, induced rupture of the filament into droplets, or induced contraction. However, the nozzle can also erase defects from the original line, such as roughness. In this work, we present strategies to ensure good fusion between filaments in EMB3D without introducing new defects. Notably, the spacing between lines, the ink rheology, the support rheology, and the interfacial tension can all influence fusion quality. For oil-based inks printed into water-based supports, many of these defects scale with the interfilament spacing and the capillary number. In many material systems it is necessary to print at a tighter spacing than ideal, leading to over-extrusion. Thus, careful selection of the material system and part design is necessary to ensure high-quality, reliable prints.

**Reference**

1. Friedrich, L.M.; Gunther, R.T.; Seppala, J.E. Suppression of Filament Defects in Embedded 3D Printing. *ACS Appl. Mater. Interfaces* **2022**, *14*, 32561–32578. https://doi.org/10.1021/acsami.2c08047.

### 11.2. Nanocalorimetry to Support In Situ Thermographic Temperature Measurements of Laser-Based Additive Manufacturing

**Brandon Lane, Jordan Weaver and Feng Yi**

National Institute of Standards and Technology
* Correspondence: pasalin@sandia.gov

**Abstract:** Laser powder bed fusion (LPBF) is a metals additive manufacturing process that uses a scanned high power laser beam to selectively melt and solidify layers of metal powder into freeform geometric shapes. The focused laser spot, typically <100 μm diameter, creates a small, molten metal pool that undergoes rapid solidification. In-situ high speed thermography is a common approach to quantify various thermal physics of the melt pool including melt pool geometry, thermal gradients, and cooling rates [1]. However, surface emissivity must be known to extract surface temperature values from the thermographic images. A common approach is to identify a discontinuity in the measured melt pool thermal profile which represents solidification, ascribe an assumed solidification temperature (often from low-rate calorimetric reference data), and use this to derive a single emissivity value. Due to the true nature of real high-rate, nonequilibrium solidification of LPBF melt pools, and known dependence of emissivity on temperature and solid/liquid phase change, using a single-value emissivity assumption will induce errors in measured melt pool temperatures. To investigate the effects of high-rate solidification on surface emissivity, the authors performed a set of thermographic measurements on the NIST nanocalorimeter apparatus capable of measuring fast physical and chemical reactions with high sensitivity [2]. Two Al7xxx alloy thin film samples were sputtered over nanocalorimeters and heated past melting, then allowed to cool and solidify at rates from <103 to >104 C/s. Using the temperature output of the nanocalorimeter surface emissivity could be extracted from the thermographic images, and spatiotemporal variation in the sample surface temperature measured via the thermographic camera. Relationships between solidification temperature, cooling rate, emissivity at solidification, and spatial distribution of the sample are analyzed. Finally, a functional relationship between surface emissivity and temperature is derived. This function is then applied to in-situ, high speed thermographic measurements of laser-scanned Al7075 samples performed on a commercial LPBF system to observe the melt pool thermal field. A comparison is made between temperature profiles and measured cooling rates of the melt pool using the traditional single-value solidification-based emissivity approach, and the more physically relevant nanocalorimeter-derived temperature dependent emissivity.

**References**

1. Lane B., Heigel J., Ricker R., Zhirnov I., Khromschenko V., Weaver J., Phan T., Stoudt M., Mekhontsev, S. and Levine L., Measurements of melt pool geometry and cooling rates of individual laser traces on IN625 bare plates *Integrating Mater. Manuf. Innov*., **2020**, 9, https://doi.org/10.1007/s40192-020-00169-1.
2. Yi, F.; Friedman, L.H.; Chen, R.; LaVan, D.A. 2019 Sample pattern and temperature distribution in nanocalorimetry measurements *J. Therm. Anal. Calorim*. **2019**, *138*, 3367–3673

### 11.3. 3D Printing and Thermo-Mechanical Characterization of High-Performance Elastomers

Rigoberto Advincula *

University of Tennessee and Case Western Reserve University
* Correspondence: rca41@case.edu

**Abstract:** The use of 3D printing to create prototypes and devices from elastomeric and polymeric materials has appended the design functionality for new materials, including uses in biomedical devices, enabling rapid development; 3D printed polymers can be further classified into thermoplastics, thermosets, and elastomers based on their thermo-mechanical properties. The processability and functionality of rubbers and elastomers make it a challenge to employ 3D printing methods for additive manufacturing. The transition to a final phase or cross-linked structure in combination with the processing method results in new properties. This is more evident with those choices of 3D printing methodologies (FDM, SLA, SLS, VSP) which can make use of blended or formulated compositions. We have demonstrated the 3D printing of several high-performance elastomers: biomedical grade thermopolastic polyurethanes (TPU), silicones, and rubberized epoxies. However, 4D printing allows the design of new materials and applications based on integrating the chemistry of conversion with the printing mode. In this talk, we demonstrate the fabrication of concept objects and elastomeric actuators based on the use of biomedical grade TPU melts and extruded viscous solutions. The result is an extrudable precursor and nancomposite elastomers which can be printed via viscous extrusion printing (VEP) or VSP, then converted to an elastomeric actuating material with very high cyclic compressibility. Other work based on the use of SLA, SLS, and FDM towards high-strength silicones and nanocomposite materials will be discussed as well. We have utilized DMA, DSC, TGA, EGA, and GC-MS pyrolysis as important thermal and thermo-mechanical characterization methods.

### 11.4. Voxel Scale Characterization in Vat Photopolymerization

Jason Killgore *

National Institute of Standards and Technology
* Correspondence: jason.killgore@nist.gov

**Abstract:** Within polymer additive manufacturing, vat photopolymerization (VP) affords a compelling combination of high throughput, high resolution, and moderate part size. These qualities are enabled by advancements in high-resolution display technologies (e.g., liquid crystal display—LCD, digital light processing—DLP) to locally cure liquid resin into solid polymer. Present methods allow direct control of millions of reactions in a single print layer up to trillions of reactions in a three-dimensional part. Each reaction can result in a voxel if chemical conversion is sufficient to achieve gelation. Notably, adjacent reactions are not completely independent due to heat transfer, species diffusion, and optical non-idealities in the light engine. Thus, local characterization of interacting voxels is essential to inform fundamental understanding and empirical control of the printing process. Here, we report on the development of novel measurement techniques to interrogate few or many voxels at better-than-voxel-scale resolution. First, we investigate the use of atomic force microscopy to probe the intrinsic mechanical heterogeneity that arises in the z (through-thickness) direction in VP. The AFM provides sufficient resolution and sensitivity to validate models of layer-wise voxel interaction. Next, we modify the atomic force microscope by incorporating a DLP light engine for in situ investigations of voxel formation. By vertically oscillating the AFM tip immersed in resin in the vicinity of the photopattern, it acts as a local photo-rheometric probe capable of measuring the reaction profile in situ with high resolution. We observed modified resin viscosity, attributed to oligomer diffusion, 10 s of microns away from photopatterns with single micron width. As the reaction proceeds further, we show that our hybrid AFM/DLP system can unambiguously identify the onset of gelation on a substrate. In bulk, the light exposure dose to achieve gelation is termed critical dose Ec. The hybrid AFM allows Ec to be studied on individual voxels as a function of photopattern size, shape, and intensity. We find that bioprinting hydrogels exhibit extreme size dependence of Ec when the voxel size approaches the transport length scale, complicating high-resolution printing. Finally, expanding from few-voxel characterization to many-voxel characterization, we introduce the use of laser scanning confocal microscopy (LSCM) for high-throughput and high-resolution characterization of voxel geometry. With the high-throughput workflow, we generate data on 100,000 interacting voxels, with precise registration between digital masks and resultant printed voxels. We use the dataset to train neural network machine learning models and achieve accurate predictions of voxel interactions with micron-scale precision. Overall, advanced characterization can yield improved additive manufacturing processes with enhanced geometric and mechanical precision.

### 11.5. AM Processing Temperature Range for PEEK

Steve Sauerbrunn *

University of Delaware—CCM
* Correspondence: sauerbru@udel.edu

**Abstract:** Additive Manufacturing (AM) of thermoplastic polymers requires a temperature minimum at which the resin can be melted and extruded through the die and deposited onto the physical design. The maximum temperature range must be below the onset of the decomposition temperature. AM has been moving into high-temperature polymers in the hope of making aerospace quality parts. Thermal degradation is present between the melting point and the onset of mass loss by TGA. While this degradation may not result in a mass loss, it will lower the polymer molecular weight by chain scission. This will lower the molecular weight and lower the resulting mechanical properties. In addition, degradation can result in chain crosslinking, which can make the polymer more brittle and result in brittle failure during use. This report will demonstrate how a standard DSC test can measure this degradation region.

## 12. Session: Adsorption

### 12.1. New Kinetically Limited Linear Driving Force (KLLDF) Model for Diffusion-Limited Adsorptive Separations

**James A. Ritter, Sulaimon A. Adegunju, Pravin B. C. A. Amalra and Armin D. Ebner**

University of South Carolina
* Correspondence: ritter@cec.sc.edu

**Abstract:** The traditional linear driving force (LDF) model is commonly used to describe mass transfer rates in fixed-bed adsorption systems. The traditional LDF model is applied on the basis of the presence of slower diffusing adsorbates affecting the equilibrium of faster diffusing adsorbates in a gas mixture via their gas phase partial pressures. In other words, if the slower diffusing components have hardly entered the pore structure, why should the partial pressure of these components still outside the pore structure influence the loadings of the faster diffusing components inside the pore structure?

The objective of this work is to present a new kinetically limited linear driving force (KLLDF) model in terms of a modified extended Langmuir (EL) model that augments the equilibrium driving force in the LDF kinetic model [1]. This augmentation is such that the equilibrium loading of one component in the LDF model depends on the actual loadings of the other components in a gas mixture, not their partial pressures. At equilibrium, the KLLDF model reduces to the LDF model, leading to the same EL model predictions.

This new KLLDF model successfully captures experimental data in the literature for carbon molecular sieve (CMS) and titanium silicate (ETS-4) adsorbents. This includes predicting the intrinsic roll-up feature of the faster diffusing species even better than Fickian diffusion models. The KLLDF model successfully captures ternary and quaternary breakthrough curves of diffusion-limited experimental data very well. These breakthrough curves comprise mixtures of CO_2_, N_2_, and CH_4_ in He adsorbed on a carbon molecular sieve carbon (CMS), and were conducted at various pressures as well as at ambient temperature. When utilizing the EL model, the traditional LDF model does not do as well as the KLLDF model in predicting these breakthrough curves, most notably in that the traditional LDF model leads to depressed loadings of the faster diffusing species, thereby underpredicting their breakthrough time in all cases. The new KLLDF model performs well in predicting the behavior of two key kinetically limited binary gas separations with a CMS when using different pressure swing adsorption (PSA) cycle schedules, namely, the CO_2_-CH_4_ and CO_2_-N_2_ gas mixtures. The KLLDF closely quantitatively predicted the performance of the PSA cycles, while the LDF model failed to do so.

This new KLLDF model requires only one fitting parameter for each component in the gas mixture, i.e., the mass transfer coefficient, just as in the traditional LDF model. Hence, it should be very useful in an adsorption process simulator. Moreover, in conjunction with the EL model for mixed gas adsorption equilibria predictions, the KLLDF model is perhaps the more appropriate model to use for any multicomponent adsorbate–adsorbent systems, whether diffusion limited or not, as it reduces to the LDF model for systems that do not exhibit significant diffusional limitations.

**Reference**

1. Adegunju, S.A.; Ebner, A.D.; Ritter, J.A. Kinetically Limited Linear Driving Force Model for Diffusion-Based Adsorptive Separations. *Ind. Eng. Chem. Res*. **2022**, *61*, 17615–17630.

### 12.2. Nanocalorimetry for Carbon Capture Materials Characterization

Feng Yi *, Avery Baumann, Arden Dombalagian, Lakshmi Narayan, John Hoffman, Christopher Stafford and David LaVan

National Institute of Standards and Technology
* Correspondence: feng.yi@nist.gov

**Abstract:** Nanocalorimetry is a nanoscale thermal measurement metrology able to explore the thermal behavior of materials at faster rates and smaller sizes, including the adsorption/desorption process. This rapid chip-based thermal measurement allows repeated cycles on the millisecond to second time scale in order to probe the thermal stability and kinetics of materials during the adsorption–desorption process. In this work, the test sample is a mixture of polyethyleneimine and silica nanoparticles. Conventional Thermogravimetric Analysis (TGA) was first used to quantify the ratio of polyethyleneimine and silica in the sample. Nanocalorimetry was then used to measure the thermal signatures of the adsorption and desorption events to help select suitable sample activation procedures. Polarization modulation infrared reflection absorption spectroscopy was used to elaborate and correlate the thermal signatures. Preliminary data show that nanocalorimetry could significantly accelerate thermal stability evaluation during the adsorption–desorption process.

### 12.3. New Kinetically Limited Linear Driving Force (KLLDF) Model for Diffusion-Limited Adsorptive Separations

Matthias Thommes *

Friedrich-Alexander University Erlangen Nürnberg
* Correspondence: matthias.thommes@fau.de

**Abstract:** Detailed analysis of the textural properties, e.g., pore size and connectivity, of nanoporous materials is essential to identify correlations of these properties with the performance of gas storage, separation, and catalysis processes. The advances in developing nanoporous materials with uniform tailor-made pore structures, including the introduction of hierarchical pore systems, offer huge potential for these applications. Within this context, major progress has been made in understanding the adsorption and phase behavior of confined fluids, and consequently in their physisorption characterization. This enables reliable pore size, volume, and network connectivity analysis using advanced high-resolution experimental protocols coupled with advanced methods based on statistical mechanics, such as methods based on density functional theory and molecular simulation. If macropores are present, a combination of adsorption and mercury intrusion/extrusion techniques can be useful. Hence, several important recent advances in understanding the mercury intrusion/extrusion mechanism are discussed. Additionally, promising complementary techniques for characterization of porous materials immersed in a liquid phase are introduced. 

Further, we will discuss the challenges associated with assessing surface chemistry characteristics (e.g., hydrophobicity/hydrophilicity). Within this context, we will present a comprehensive strategy based on the combination of adsorption, liquid intrusion, and NMR-based techniques for quantitative assessment of the hydrophilicity/hydrophobicty of pore surfaces.

### 12.4. Reference Adsorption Isotherms Using Reference Materials

Roger van Zee *

National Institute of Standards and Technology
* Correspondence: roger.vanzee@nist.gov

**Abstract:** Adsorbent materials have many applications, including those related to gas storage, gas purification, catalytic reforming, and sustainable development. Despite major progress in adsorption technology and physical adsorption characterization during the past two decades, measurement challenges still exist. For example, protocols for measuring high-pressure gas adsorption isotherms on well-characterized porous materials have not been standardized. To address these measurement needs, the Facility for Adsorbent Characterization and Testing (“FACT Lab”) at the National Institute of Standards and Technology has undertaken a series of interlaboratory studies to measure and quantify adsorption isotherms using reference materials. Reference isotherms of carbon dioxide adsorption on ZSM-5 zeolite (NIST Reference Material 8852) have been determined, including as a function of temperature, to 4.5 MPa. Additionally, a reference isotherm for methane adsorption on zeolite y (NIST Reference Material 8850) has been determined to 7.5 MPa. Simple empirical functions describing these isotherms were derived. More recently, isotherms for water adsorption on a nanoporous carbon (BAM Certified Reference Material P-109) have been determined to 95% relative humidity and for neat water. Future work will focus on adsorption of binary gas mixtures on zeolites; preliminary results will be presented.

### 12.5. Using the Heat of Immersion to Quantify Contact Angle: A Re-Examination

Brian Grady * and Jason Xu

University of Oklahoma
* Correspondence: bpgrady@ou.edu

**Abstract:** Previous work [1,2,3] showed that the heat of immersion method was excellent for measuring wetting of powders, with a more negative heat corresponding to better wetting. In this paper, this work is extended experimentally to higher temperatures. One advantage of heat of immersion is that measuring at pressure above atmospheric is straightforward, allowing quite a large range of temperatures to be investigated. Finally, as will be seen, the measured contact angles are incorrect. Hence, the derivation is revisited and the limitations of this approach are clearly delineated. 

**References**

1. Yan, N.X.; Maham, Y.; Masliyah, J.H.; Gray, M.R.; Mather, A.E. Measurement of contact angles for fumed silica nanospheres using enthalpy of immersion data. *J. Colloid Interface Sci.* **2000**, *228*, 1–6.
2. Spagnolo, D.A.; Maham, Y.; Chuang, K.T. Calculation of contact angle for hydrophobic powders using heat of immersion data. *J. Phys. Chem.* **1996**, *100*, 6626–6630.
3. Weston, J.S.; Jentoft, R.E.; Grady, B.P.; Resasco, D.E.; Harwell, J.H. Silica nanoparticle wettability: Characterization and effects on the emulsion properties. *Ind. Eng. Chem. Res.* **2015**, *54*, 4274–4284.

## 13. Session: Student Posters

### 13.1. Taguchi Method-Based Evaluation of Thermal Hazard of Lift-Off Pyrotechnics under Various Storage Conditions

Shin-Mei Ouyang, Yia-Chi Ye * and Wei-Chun Chen

National Yunlin University of Science and Technology
* Correspondence: in16882@gmail.com

**Abstract:** This study employed the Taguchi method and analysis of variance to investigate the factors that influence the deterioration of the lift-off pyrotechnics commonly used in celebration events in Taiwan when they are stored under various relative humidities (RH) and temperature conditions. A field emission scanning electron microscope and an energy-dispersive X-Ray spectroscope were used to observe the microstructures and elements of pyrotechnic samples. Differential scanning calorimetry (DSC) and vent sizing package 2 (VSP2) were applied to measure the relationship between the trace heat flow of the samples and temperature as well as the thermal runaway reactions in a pseudo-adiabatic environment. A DSC analysis and a thermokinetic ASTM E698 analysis were combined to explore the safety of lift-off pyrotechnics and the related thermal hazards.

The energy-dispersive X-Ray spectroscopy and DSC experimental results revealed that KNO3 is highly soluble. However, when RH and exposure time increased, the surface dissolution of oxidants reduced the second exothermic peak, resulting in incomplete combustion and ignition failure. The ASTM E698, thermokinetic analysis results indicated that the apparent activation energy (Ea) of the samples increased substantially after they were immersed in deionized (DI) water. Notably, the Ea of color smoke type increased from 46.9 to 155.9 kJ mol^−1^, indicating that the examined pyrotechnics were more sensitive to a decrease in temperature than to an increase in RH. A large amount of water can be used to alleviate the reactivity of pyrotechnics and thereby diminish pyrotechnic hazards. The VSP2 analysis results indicate that the self-boosting rate of the samples was greater under 40% RH than under pristine conditions; however, this rate decreased substantially under 80% RH and after immersion in DI water. The results of the Taguchi experiment and analysis of variance revealed that RH contributed 63.60% and 50.86% to Ea and maximum pressure, respectively. Accordingly, governing RH should be prioritized in measures for diminishing pyrotechnic hazards.

### 13.2. Thermal, Structural, and Surface Analysis of Electrospun Nanofiber Membranes from Polyampholyte/Poly(vinylidene fluoride) Blends

Anuja Jayasekara *, Luca Mazzaferro, Ayse Asatekin and Peggy Cebe

Tufts University
* Correspondence: anuja.jayasekara@tufts.edu

**Abstract:** Poly (vinylidene fluoride) (PVDF) is a material used extensively in fabricating filtration membranes due to its high thermal and chemical resistivity, low cost, and low surface energy density [1]. However, due to its excellent hydrophobicity, PVDF membranes have a high tendency to foul during the filtration process [2]. Incorporating hydrophilic substances into the PVDF membranes is a broadly used method to reduce membrane fouling [2]. Polyampholytes are large molecules that contain separate positively and negatively charged side groups on their backbone [3]. Due to these charged subunits, polyampholytes show high hydrophilicity, leading them to exhibit anti-fouling properties useful in filtration applications [4], including their blends with PVDF [5]. However, to the best of our knowledge, no studies have yet been performed on PVDF/polyampholyte blends prepared as electrospun fibers. Here, we electrospun nanofibrous membranes from a random polyampholyte copolymer (r-PAC) blended with PVDF. The r-PACs are composed of monomers that are either hydrophobic (2,2,2-trifluoroethyl methacrylate), positively charged ([2-(methacryloyloxy) ethyl]trimethylammonium chloride), or negatively charged (methacrylic acid). In the electrospun membranes, the hydrophilic units of r-PAC provide the fouling resistance and the hydrophobic units in both r-PAC and PVDF provide the mechanical and chemical stability. Solutions of PVDF/r-PAC with different compositions were prepared by controlling the ratio of PVDF to r-PAC. The electrospun fiber mats were characterized for their structure using wide-angle X-Ray scattering. Degradation temperature and glass transition properties were evaluated using thermogravimetric (TG) analysis and differential scanning calorimetry, respectively. TG results of homopolymer r-PAC, both the as-received material and the electrospun fibers, show two degradation steps at 250 °C and 450 °C. A similar trend was seen in blends of PVDF/r-PAC prepared as electrospun fiber mats. The blended fiber mats with large PVDF content showed an additional degradation step corresponding to the degradation of C-F bonds in the PVDF component. Sessile drop contact angle measurements were performed to evaluate the surface wettability of membranes for water and oil (dodecane).

**References**

1. Zhou, Z.; Wu, X.-F. Electrospinning superhydrophobic–superoleophilic fibrous PVDF membranes for high-efficiency water–oil separation. *Mater. Lett*. **2015**, *160*, 423–427.
2. Li, Y.S.; Yan, L.; Xiang, C.B.; Hong, L.J. Treatment of oily wastewater by organic–inorganic composite tubular ultrafiltration (UF) membranes. *Desalination* **2006**, *196,* 76–83.
3. Dobrynin, A.V.; Colby, R.H.; Rubinstein, M. Polyampholytes. *J. Polym. Sci. Part B Polym. Phys.* **2004**, *42,* 3513–3538.
4. Neitzel, A.E.; De Hoe, G.X.; Tirrell, M.V. Expanding the structural diversity of polyelectrolyte complexes and polyzwitterions. *Curr. Opin. Solid State Mater. Sci*. **2021**, *25,* 100897.
5. Shen, X.; Yin, X.; Zhao, Y.; Chen, L. Antifouling enhancement of PVDF membrane tethered with polyampholyte hydrogel layers. *Polym. Eng. Sci* **2015**, *55,* 1367–1373.

### 13.3. Impact of Al_2_O_3_ Nanoparticle Reinforcement on Polysulfone

Nazanin Shakoury * and Gregory McKenna

North Carolina State University
* Correspondence: nshakou@ncsu.edu

**Abstract:** Polysulfone (PSF) is among the few polymers that have a weak β-relaxation and a strong γ-relaxation [1]. These secondary transitions are associated with properties such as elongation and impact strength; thus, including fillers in the polymer matrix has the potential for plasticization, reinforcement, or antiplasticization (fortification) by interaction with the β -or γ-relaxations [2]. There have been different studies investigating the effect of nanoparticles on the sub-glass relaxations as well as on the glass transition temperature (Tg), indicating that both negative and positive changes in Tg are possible [3,4]. In this research, we investigate the impact of Al2O3 nanoparticles on the thermal behavior and secondary loss transitions of PSF, expecting that the interactions of the nano-sized alumina and PSF can lead to different phenomena. Strong attractive interactions between nanoparticles and the host polymer provide the possibility of in antiplasticization and reinforcement, while plasticization is the outcome of a weak interaction between the filler and polymer [5]. Furthermore, confinement effects on the matrix due to the near neighbor spacing of the nanoparticle dispersions can impact the polymer dynamics as well [6].

Here, we report the influence of different concentrations of 13 nm diameter alumina nanoparticles dispersed in PSF on the glass transition temperature of the PSF using differential scanning calorimetry (DSC). Samples were prepared by the solvent casting of PSF in dichloromethane (DCM) filled with nanoparticles. After mixing PSF and DCM, nanoparticles were added followed by sonication of the mixture to ensure good particle dispersion. The samples were dried for 24 h in a fume hood at room temperature followed by DSC characterization.

Preliminary results suggest that the Al_2_O_3_ nanoparticles have little impact on the glass transition temperature of the polysulfone for particle concentrations from 1% to 10% alumina, though one series of tests did show a decrease in the Tg of about 4 K for the sample containing 1 mass % of the Al_2_O_3_, while other concentrations of the nanoparticles showed virtually no change in the Tg. Further work is ongoing to determine the origins of the effect should it be reproducible.

In other work, we are characterizing the impact of the nanoparticle addition on the viscoelastic moduli (dynamics) of the polymer, including both above and below the glass transition temperature, with the impact of the Al2O3 additives on the sub-glass b- and g- relaxations.

**References**

1. Fried, J.R.; Letton, A.; Welsh, W.J. Secondary relaxation processes in bisphenol-A polysulphone. *Polymer* **1990**, *31*, 1032–1037. https://doi.org/10.1016/0032-3861(90)90249-X.
2. Robeson, L.M.; Faucher, J.A. Secondary loss transitions in antiplasticized polymers. *J. Polym. Sci.* **1969**, *7*, 35–40. https://doi.org/10.1002/pol.1969.110070108.
3. Yim, A.; Chahal, R.S.; Pierre, L.E.S. The effect of polymer—filler interaction energy on the T′ g of filled polymers. *J. Colloid Interface Sci.* **1973**, *43*, 583–590. https://doi.org/10.1016/0021-9797(73)90406-2.
4. Ash, B.J.; Siegel, R.W.; Schadler, L.S. Glass-transition temperature behavior of alumina/PMMA nanocomposites (vol 42, pg 4371, 2004). *J. Polym. Sci. Part B: Polym. Phys.* **2005**, *43*, 114. https://doi.org/10.1002/polb.20333.
5. Comer, A.C.; Heilman, A.L.; Kalika, D.S. Dynamic relaxation characteristics of polymer nanocomposites based on poly (ether imide) and poly (methyl methacrylate). *Polymer* **2010**, *51*, 5245–5254. https://doi.org/10.1016/j.polymer.2010.08.053.
6. Alcoutlabi, M.; McKenna, G.B. Effects of confinement on material behaviour at the nanometre size scale. *J. Phys. Condens. Matter* **2005**, *17*, R461. https://doi.org/10.1088/0953-8984/17/15/R01.

### 13.4. Hazard Investigation of Hydrocarbon Solvent in Ultrasonic Cleaning Processes

Chun-Yen Tseng, Yia-Chi Ye * and Wei-Chun Chen

National Yunlin University of Science and Technology
* Correspondence: in16882@gmail.com

**Abstract:** This study investigated the safety and potential hazards of using hydrocarbon solvents in circulation during ultrasonic cleaning processes. The recycling and reuse of solvents, which includes procedures such as ultrasonic agitation, vacuum boiling, and distillation condensation, may result in the formation or breakdown of molecules and alterations to the structure or properties of the solvent. Furthermore, the solvent may become contaminated with impurities, such as dirt, oil, grease, and metal particles, during the ultrasonic cleaning process, leading to changes in the flash point and flammability limit of the solvent as well as expanding the range of explosion. The process conditions cause the recycled solvent to change its properties, making it more difficult to predict potential hazards in its use.

This study confirmed that recirculation of hydrocarbon solvent in ultrasonic cleaning processes poses a risk of fire and explosion; therefore, strict monitoring and close attention to process operating conditions should be carried out during the ultrasonic cleaning process to ensure the safety of the hydrocarbon solvent. This will help reduce potential hazards in industrial cleaning processes and provide a safe and healthy environment for plant operators.

### 13.5. The Glass Transition in Indomethacin/Sucrose Benzoate Co-Amorphous Drug Delivery Systems

Elaheh A. T. Moghadam * and Sindee Simon

North Carolina State University
* Correspondence: eabdoll@ncsu.edu

**Abstract:** The glass transition temperature (Tg) and related dynamics are investigated for binary miscible mixtures of indomethacin and sucrose benzoate using differential scanning calorimetry. These mixtures are termed co-amorphous glasses; such materials have been shown to possess enhanced water solubility and bioavailability relative to the crystalline forms of active pharmaceutical ingredients. For the co-amorphous glasses studied here, the composition-dependent Tg displays negative deviations from expectations for an athermal mixture, and the data cannot be described by the Fox equation. The ability of other approaches to describe composition-dependent Tg will be discussed. In addition, the cooling rate dependence of the Tg and the fragility of each mixture is determined in an effort to clarify the relationship between fragility, ease of glass formation, and stability of the glass against crystallization.

### 13.6. Effects of Chemical Structure Variation and Salt Addition on the Crosslinking of Polyzwitterion/LiCl Complexes

**John Thomas, Peggy Cebe, Abhishek Mondal and Ayse Asatekin**

Tufts University
* Correspondence: john.thomas@tufts.edu

**Abstract:** Polyzwitterions (PZIs) have a wide range of applications, such as solid polymer electrolytes, filtration membranes, and drug delivery. To build a knowledge base on the solid-state properties of these polymers, the following PZIs were studied: poly(sulfobetaine methacrylate), PSBMA; poly(sulfobetaine acrylate), PSBA; and poly(ethyl sulfobetaine methacrylate), PESBMA. PZIs have covalently linked anions and cations on the same chain, creating strong intra- and interchain dipole–dipole electrostatic crosslinks. Through the addition of LiCl and variations in the monomeric chemistry, the formation of these crosslinks can be hindered. Due to the disruption of crosslinking, there are changes in the thermal degradation temperature, glass transition temperature (Tg), and the solid and liquid state specific heat capacities. These PZI/LiCl complexes are hydrophilic and hygroscopic, imparting significant plasticization effects which must be removed in order to isolate the effects of chemical structure variations and salt addition. Using thermogravimetric analysis (TGA) and temperature modulated DSC (TMDSC), we can analyze the bound water content in PZI/LiCl complexes as a function of change in mass during the first heating. Using long isothermal holds at low temperatures followed by successive heating to high temperatures, surface- and molecularly-bound water can be consistently removed. When the dry state is reached, we find that as the molar ratio of LiCl increases, the crosslinking density decreases, the glass transition is lowered, and the heat capacity increment at the glass transition increases. This change in the heat capacity increment is due to nonconfinement of the complex through the breaking of crosslinks. The effects on the glass transition process closely resemble the confinement seen in semi-crystalline polymers. We used Wunderlich’s bead-approximation to estimate the contribution of freely rotatable bonds to the heat capacity increment at Tg for these complexes, and found that at high salt ratios the reduction in confinement allowed the system to reach the freely rotatable state.

## 14. Session: General Posters

Improving Thermal Stability and Flammability Limit on Paraffin Wax by Mixing with Phosphorus-Containing Ionic Liquid

Yu-Cheng Lin, Wei-Cheng Lin, Yi-Chun Yu and Chi-Min Shu *

National Yunlin University of Science and Technology
* Correspondence: shucm@yuntech.edu.tw

**Abstract:** Paraffin is often used as an additive in numerous products; however, many cases of fire are closely related to its flammability. The prevention of fires caused by paraffin can be divided into two aspects: first, focusing on the use of fire in order to decrease the frequency of fires; and second, ameliorating the flame-retardant characteristics of paraffin. Ionic liquids (ILs) are often used as flame retardants due to their non-volatility, sound thermal stability, and relative low flammability limit, and among them phosphorus-containing ILs are the most notable in support of flame retardancy. In this study, 1-butyl-3-methylimidazolium dibutylphosphate ([Bmim][DBP]) and 1-butyl-3-methylimidazolium hexafluorophosphate ([Bmim][PF6]) were employed. To mix ILs in paraffin, adding diatomite substantially improved the binding ability of paraffin and ILs when the paraffin was in the liquid state. The pure paraffin and paraffin phosphorus-containing ILs obtained after solidification were treated as samples for subsequent analysis and testing.

Using simultaneous thermogravimetric analyzer (STA), paraffin mixed with diatomite exhibited the highest heat flow value (10.23 W/g). On the other hand, after mixing paraffin with [Bmim][DBP] and [Bmim][PF6], it was able to release lower heat and high heat flow values (4.42 and 4.51 W g^–1^, respectively) in a high-temperature environment. Based on thermogravimetric analysis (TGA), the paraffin containing ILs showed a lower thermal peak, and the apparent onset temperature of the pyrolysis zone (To) and final temperature of the thermal decomposition zone (Tf) were both delayed from that of pure paraffin. According to the above methods, the paraffin composed of 8.0 mass% [Bmim][DBP] and 4.0 mass% [Bmim][PF6] embodied the optimal flame-retardant properties.

Furthermore, the Fourier-transform infrared spectroscopy (FTIR) was applied to analyze the evolved products for paraffin phosphorus-containing ILs during thermal degradation. The observed peaks at wavenumbers 2300 and 2900 cm^–1^ were carbon dioxide (O=C=O) and alkanes (C-H), respectively. Finally, proper thermokinetic models were applied to determine its apparent activation energy (Ea). It was predicted that paraffin containing 8.0 mass% [Bmim][DBP] and 4.0 mass% [Bmim][PF6] was the optimal addition ratio.

